# A Scalable Approach for Discovering Conserved Active Subnetworks across Species

**DOI:** 10.1371/journal.pcbi.1001028

**Published:** 2010-12-09

**Authors:** Raamesh Deshpande, Shikha Sharma, Catherine M. Verfaillie, Wei-Shou Hu, Chad L. Myers

**Affiliations:** 1 Department of Computer Science and Engineering, University of Minnesota - Twin Cities, Minneapolis, Minnesota, United States of America; 2 Department of Chemical Engineering, University of Minnesota - Twin Cities, Minneapolis, Minnesota, United States of America; 3 Interdepartmental Stem Cell Institute Leuven, Catholic University Leuven, Leuven, Belgium; New York University, United States of America

## Abstract

Overlaying differential changes in gene expression on protein interaction networks has proven to be a useful approach to interpreting the cell's dynamic response to a changing environment. Despite successes in finding active subnetworks in the context of a single species, the idea of overlaying lists of differentially expressed genes on networks has not yet been extended to support the analysis of multiple species' interaction networks. To address this problem, we designed a scalable, cross-species network search algorithm, neXus (Network - cross(X)-species - Search), that discovers conserved, active subnetworks based on parallel differential expression studies in multiple species. Our approach leverages functional linkage networks, which provide more comprehensive coverage of functional relationships than physical interaction networks by combining heterogeneous types of genomic data. We applied our cross-species approach to identify conserved modules that are differentially active in stem cells relative to differentiated cells based on parallel gene expression studies and functional linkage networks from mouse and human. We find hundreds of conserved active subnetworks enriched for stem cell-associated functions such as cell cycle, DNA repair, and chromatin modification processes. Using a variation of this approach, we also find a number of species-specific networks, which likely reflect mechanisms of stem cell function that have diverged between mouse and human. We assess the statistical significance of the subnetworks by comparing them with subnetworks discovered on random permutations of the differential expression data. We also describe several case examples that illustrate the utility of comparative analysis of active subnetworks.

## Author Summary

Microarrays are a powerful tool for discovering genes whose expression is associated with a particular biological process or phenotype. Differential expression analysis can often generate a list of several hundred or even thousands of significant genes. While these genes represent real expression differences, the large number of candidates can make the process of hypothesis generation for further experimental studies challenging. Use of complementary datasets such as protein-protein interactions can help filter such candidate lists to genes involved with the most relevant pathways. This approach has been applied successfully by many groups, but to date, no one has developed an approach for discovering active pathways or subnetworks that are conserved across multiple species. We propose an algorithm, neXus (Network – cross(X)-species – Search), for cross-species active subnetwork discovery given candidate gene lists from two species and weighted protein-protein interaction networks. We validate our approach on expression studies from human and mouse stem cells. We find many active subnetworks that are conserved across species relevant to stem cell biology as well as other subnetworks that show species-specific behavior. We show that these networks are not likely to have been discovered by chance and discuss several specific cases that reveal potentially novel stem cell biology.

## Introduction

Developments in genomic and proteomic technologies in recent years have given us numerous methods for capturing high resolution snapshots of cellular processes. The end result of a genome-scale experiment is typically a long list of candidate genes that provide a basis for further, more detailed, follow up experiments. For example, gene expression microarrays are a popular approach for identifying differentially expressed genes between two cell types or experimental conditions, and this technology typically yields several hundred to a few thousand differentially expressed genes in a typical comparison [1], [2]. While there are sometimes obvious biological processes represented within these lists, developing precise hypotheses from such a long list of candidates can be challenging. Although to varying degrees, this is also true of other genome-scale experiments or screens (e.g. Genome wide association studies [3] or genetic interaction screens [4]). In short, the bottleneck in genomic research has quickly moved from the production of high-quality data to interpretation and hypothesis generation.

One powerful approach that has been used to aid in the interpretation of candidate genes lists is integrative analysis with complementary genome-scale data. For example, in a landmark study, Ideker *et al.* addressed the challenge of interpreting lists of significantly differentially expressed genes by overlaying them on a protein-protein interaction network [5]. They found that certain groups of differentially expressed genes tend to cluster together on the interaction network, building confidence that the signature was indeed biologically relevant and suggesting that entire physical modules were differentially expressed together. This approach has since been extended to several other scenarios, all demonstrating the utility of this idea. For example, Rajagopalan *et al.* extended Ideker's method to larger, literature-curated biological networks [6]. Others incorporated co-expression scores to favor selected edges of the protein interaction network [7], [8], [9], [10]. Dittrich *et al.* later formulated the problem as an integer linear programming optimization problem [10]. Recent work has also extended this idea to show that sample classification based on expression profiles can also take advantage of complementary structural information in protein-protein interaction networks [11].

In separate studies, groups have compared and aligned the structure of protein-protein interaction networks across species [12], [13]. The basic approach adopted by these methods is to identify subgraphs with conservation at the protein sequence level (nodes) as well as at the physical or functional interaction level (edges). This approach has been used to suggest core pathways that are conserved across species and to build confidence in individual protein-protein interactions based on the co-occurrence in multiple species [12], [13]. However, to our knowledge, no one has yet applied this idea to study network-based patterns of expression across species. We propose that just as protein-protein interaction networks can be mined for conserved patterns, differential expression patterns overlaid on biological networks can be aligned to identify conserved patterns of expression, which we call *conserved active subnetworks*.

In this study, we describe a novel approach for identifying conserved active subnetworks in interaction networks across multiple species. Given differential expression measures representing analogous phenotypes in two different species and corresponding interaction networks (for example, protein-protein interaction networks), our approach identifies tightly connected network modules that show a high degree of differential expression, i.e. dense subnetworks, and are conserved in both networks. This is in contrast to previous approaches, which focused on using differential expression or other activity scores to identify dense subnetworks in protein-protein interaction networks for a single species [5], [6], [7], [8], [9], [10], [11].

In addition to addressing the new question of conservation of network patterns across species, our approach presents a scalable solution to active subnetwork identification, which has typically been restricted to relatively sparse protein-protein interaction networks. Sparse coverage of current protein-protein interaction studies limits the ability to match patterns across species. Recent work in area of genomic data integration helps to address this issue. Several approaches now exist which integrate interaction and other information to infer functional associations between genes, to form functional linkage networks [14], [15], [16]. Such approaches can incorporate protein-protein and genetic interactions, gene expression, protein localization, phenotype, and sequence data; and have been applied now in many species including yeast, bacteria, worm, fly, plants (Arabidopsis), mouse, and human [14], [15], [16], [17], [18], [19], [20], [21]. These networks are often significantly denser than protein-protein interaction networks and include hundreds of thousands or even millions of weighted edges that reflect confidence in gene-gene functional relationships. The power (and challenge) in using functional linkage networks is that they capture a broad range of functional relationships that have relevance for defining network modules: for example, physical interactions between proteins, co-expression, regulatory relationships, or shared mutant phenotypes. This is in contrast to protein-protein interaction networks which focus on physical interactions between proteins, our knowledge of which is relatively limited in many species, particularly higher eukaryotes. A more detailed comparison of functional linkage and protein-protein interaction networks and the implications for their use for active subnetwork discovery is provided as Supplementary Material (see a detailed discussion in Text S1, Note 1, “Implications of using functional linkage vs. physical interaction networks for active subnetwork discovery”).

Given their more comprehensive coverage of a broad variety of gene relationships, functional linkage networks should allow for more sensitive discovery of networks that are differentially expressed under various conditions of interest. However, with their broader coverage also come several computational issues. Given the fact that functional linkage networks are orders of magnitude more dense than protein-protein interaction networks, existing algorithms for the discovery of dense subnetworks do not easily scale to this problem. Using functional linkage networks from human and mouse as a basis, we applied our scalable cross-species network discovery approach to identify conserved subnetworks that are differentially active in stem cells relative to differentiated cells based on parallel gene expression studies in mouse and human. We show that these conserved patterns are not likely to have occurred by chance, and that they are enriched for known as well as novel stem cell and differentiation-related processes. Another useful application of our approach is to find functional modules which have diverged or which have been rewired across the two species, which has been previously approached using expression data alone [22]. We designed a variation of our cross-species network search approach to find a number of species-specific networks, which likely reflect differences in the active cellular program between mouse and human pluripotent stem cells. Finally, we demonstrate the usefulness of our algorithm by discussing specific examples of subnetworks discovered, some of which highlight the potentially novel candidate genes involved in the maintenance of stem cell pluripotency.

## Results/Discussion

### A method for discovering conserved active subnetworks across species

**Figure 1:**
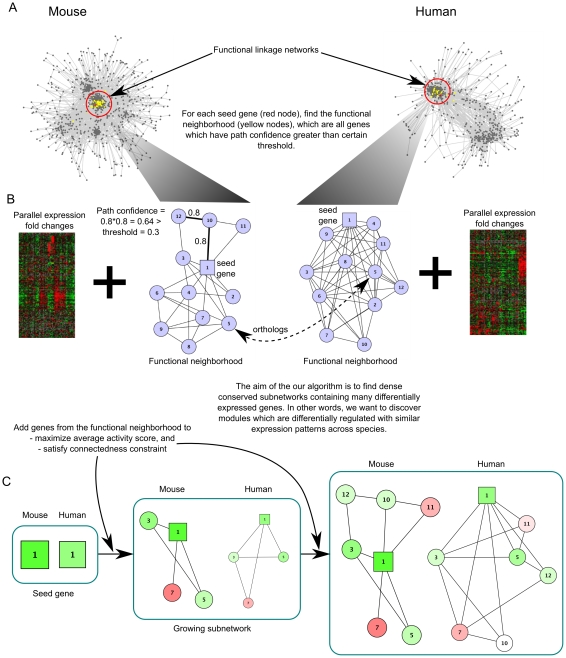
A method for discovering conserved active subnetworks across species. (A) The flowchart describes the growth of a subnetwork from a candidate seed gene (red) in the functional linkage network. (B) Genes that are functionally related to the seed are defined as those whose path confidence from the seed gene is above a certain threshold (colored yellow in A), and are considered to be the functional neighborhood of the seed. The aim of the approach is to integrate the expression data with functional linkage networks and discover active conserved subnetworks. (C) The candidate subnetwork initially contains the seed gene and is grown by adding genes iteratively from the functional neighborhood so as to maximize the average expression activity score of the genes in the subnetwork. At all iteration steps, the connectivity constraint must be satisfied before a candidate gene is added. The nodes in the growing subnetworks are genes and the edge-weights are derived from the functional linkage network in either species. The genes are colored green if they are up-regulated in stem cells relative to differentiated cells and red if they are down-regulated in stem cells relative to differentiated cells. The color intensity represents the expression normalized fold change in either direction.

We developed an algorithm to find conserved active subnetworks across species (Figure 1). Our approach requires lists of differentially expressed genes and corresponding fold change values in two different species, assumed to represent analogous conditions. The aim of our approach is to overlay gene activity scores on the respective functional linkage or interaction networks to discover dense subnetworks with a large number of differentially active genes with similar expression patterns in both species. Our approach assumes a set of orthologous clusters for the two species of interest and weighted linkage networks in both species, although it can be also applied to binary interaction networks (e.g. protein-protein interaction networks [23]). Briefly, subnetworks are simultaneously grown in both species from seed genes by adding nearby genes in the interaction networks that maximize the average activity score of the subnetwork while at the same time maintaining a minimum desired clustering coefficient of the genes in the subnetwork (see Materials and Methods for details). Subnetwork growth is stopped when the average activity score reaches a minimum threshold. This process is then repeated with each differentially active gene in either species serving as the seed. The result is a set of highly clustered subnetworks with a high density of matched differential expression in both species (see Materials and Methods for details).

### Differential expression analysis of a compendium of human and mouse stem cell expression data

To test our subnetwork discovery method, we compiled a compendium of gene expression data for mouse and human pluripotent stem cells. Briefly, 249 mouse and 132 human expression profiles were obtained from several independent datasets from the Gene Expression Omnibus (GEO) database [24] (Table S3, S4). Our goal was to identify subnetworks whose activity was associated with the maintenance of stem cell pluripotency in both human and mouse. It has been shown that human embryonic stem (ES) lines across the world are identical in expression of key pluripotency markers like Nanog and Pou5f1, but they can show remarkable differences in expression of other lineage specific markers such as AFP, possibly due to different culture conditions and varying levels of spontaneous differentiation in cultures [25]. Thus, we reasoned that a large compendium of data in both species could support a more robust differential expression analysis, free of any biases from individual studies or cell lines. To group expression profiles at similar stages of differentiation, we used non-negative matrix factorization (NMF) [26], which is an unsupervised clustering method (see Materials and Methods for details). Clusters resulting from NMF clearly separated the expression profiles of undifferentiated, pluripotent cells from those that were in early stages of differentiation or late stages of reprogramming. Differential expression analysis (SAM) was then performed between these two classes of samples to identify a set of genes that change in expression as the pluripotent cells start to exit the self-renewal program during differentiation (see Materials and Methods for details). This clustering and differential expression analysis process was performed independently on the mouse and human expression data. The genes deemed significant by this analysis were labeled with activity scores reflecting normalized fold change values (see Materials and Methods for details) and used as input for our subnetwork discovery approach.

It is important to note that the method for differential expression analysis (or other means of generating activity scores) is completely independent of the subnetwork discovery algorithm. Our large compendium of stem cell expression data for mouse and human provided an interesting setting for subnetwork discovery, but our approach could also be applied to activity scores derived from more standard, single-dataset differential expression studies, assuming comparable datasets are available for two different species (see Text S1, Note 2, “neXus applied to single dataset differential expression study” and Figure S1 for an example).

### Evaluation of conserved subnetworks

We applied our subnetwork discovery approach to the results of the stem cell differential expression analysis and functional linkage networks from human and mouse. Human and mouse functional linkage networks were obtained from previous work [15], [16]. The human network incorporates physical and genetic interactions, sequence information (shared protein domains, transcription factor binding sites), and gene expression profiles [15]. The mouse network incorporates physical interaction data, shared phenotype data, phylogenetic profile information, the yeast functional linkage network where orthologs exist, and gene expression information [16]. These functional networks reflect broad functional relationships between genes or proteins and thus are more general than protein-protein interaction networks (see a detailed discussion in Text S1, Note 1, “Implications of using functional linkage vs. physical interaction networks for active subnetwork discovery”). While the input data for these networks are largely independent, physical interaction data for mouse was derived from human interactions (see a detailed discussion in Text S1, Note 3, “Independence of the datasets”).

**Figure 2:**
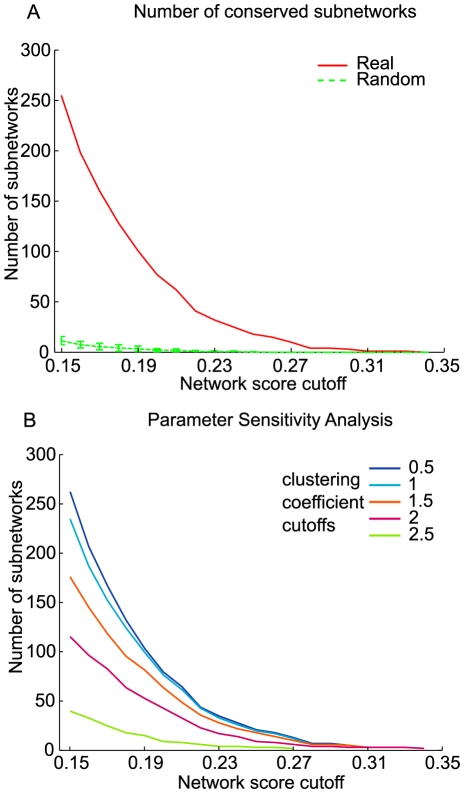
Evaluation of conserved subnetworks. (A) The cross-species algorithm mines subnetworks in the functional linkage network with a high density of differentially expressed genes. The network score of a subnetwork reflects the average differential activity of all genes in the network. The number of subnetworks identified at a network score threshold is plotted (solid line) and is compared to the number of subnetworks identified after differential expression scores were randomly shuffled (dotted line). The parameters for average clustering coefficient are 0.1 for mouse and 0.2 for human. (B) The number of conserved subnetworks discovered is plotted for a range of connectedness parameters (minimum clustering coefficient). All clustering coefficients noted are relative to the background, single-gene average clustering coefficient, which is 0.08 for mouse and 0.35 for human.

Conserved active subnetworks between human and mouse were identified by varying the two parameters of the algorithm, the average expression activity (normalized fold change) of the network, and the minimum clustering coefficient. This resulted in between 1 and 255 network(s) from the most conservative to the most lenient parameter settings, respectively. For example, at a network score cutoff of 0.15 (see Materials and Methods, “Microarray data processing” for fold change normalization), and strict clustering coefficient criteria (>0.1 for mouse and >0.2 for human), we found a total of 255 conserved subnetworks involving 607 genes in each of the two species (Figure 2A). Increasing the clustering coefficient cutoff or increasing the network score threshold enabled the discovery of fewer, but increasingly confident subnetworks (Figure 2B, Figure S2).

To assess the statistical and biological significance of the networks, we performed a network randomization analysis. Specifically, the expression activity scores in both mouse and human were randomly shuffled five times with respect to the gene labels, and the algorithm was then applied to the shuffled expression profiles. Any conserved patterns of these randomized expression data on the functional linkage network should then represent false positives and not biologically relevant conservation. In all randomization experiments, the functional linkage network structure was retained and only gene activities were shuffled, so that we could specifically estimate the conserved expression patterns arising out of clustering of the active genes by random chance. Importantly, we found that while some subnetworks were discovered in various instances of the randomization experiment, far fewer subnetworks were discovered than for the original expression profiles (Figure 2A). For example, at our lenient network score and clustering coefficient cutoffs, we discovered an average of 11.4 subnetworks (standard deviation of 4) across five randomization experiments in contrast to the 255 real subnetworks discovered on the original expression data (Figure 2A). Moreover, the average size of the real subnetworks was much larger than the random subnetworks as they contained an average of 22 genes compared to 5.7 genes (standard deviation of 0.6) across the random trials. This comparison clearly suggests that the subnetworks obtained by our cross-species approach are statistically significant, and are not likely to have been discovered by chance. We also found that the signal to noise ratio, which is the ratio of number of real subnetworks to the average number of random subnetworks, improved as we increased the network score cutoff (Figure S3) and clustering coefficient cutoffs (Figure S2). This improvement suggests that tuning these parameters is an effective means of isolating high-confidence conserved network signatures for hypotheses generation.

**Figure 3:**
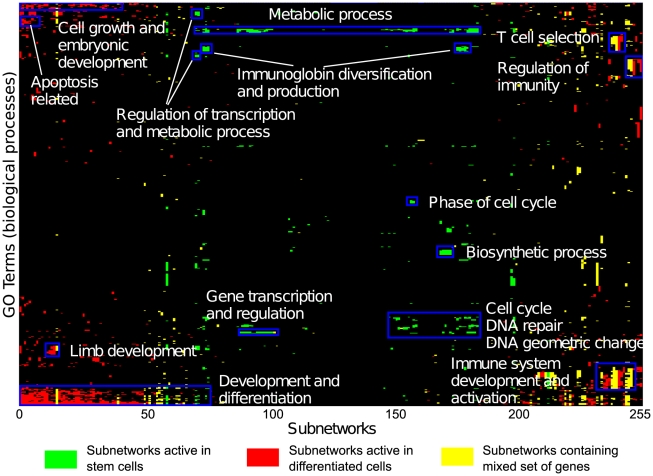
Functional summaries of the subnetworks. The 2D hierarchically clustered matrix of subnetworks' functions highlights functional enrichments based on Gene Ontology annotations (biological process category) for the mouse counterparts of all conserved active subnetworks. A subnetwork column is colored green if the subnetwork contained genes predominantly up-regulated in stem cells, red if the genes in the subnetwork are up-regulated in differentially expressed cells, and yellow, if the subnetwork contains mixed genes, some of which are more highly expressed in stem cells and some in differentiated cells. Enrichment was measured for all GO terms (Bonferroni-corrected p<0.05), and the enrichment patterns were clustered to reveal patterns of enrichment across the subnetworks. Enriched GO Terms for individual subnetworks have been uploaded on the subnetworks website and can be browsed at http://csbio.cs.umn.edu/neXus/subnetworks. The enriched GO Terms for stem cells, differentiated cells and mixed subnetworks can be found in Table S5.

We also evaluated the subnetworks in terms of their functional coverage and relevance. The function enrichment of the genes contained in each subnetwork was measured based on significant overlap with biological processes in the Gene Ontology [27] (see Materials and Methods). A large majority of the subnetworks (235 of 255) were found to be enriched for GO processes, many with suspected involvement in stem cell maintenance and differentiation (Figure 3). Furthermore, many subnetworks were monochromatic, that is, they contained genes with concordant changes in expression in either stem cells or differentiated cells. Around a third of the subnetworks were consistently more highly expressed in stem cells while approximately half of them were consistently more highly expressed in differentiated cells. As expected, the monochromatic subnetworks active in stem cells were found to play a role in metabolic processes and regulation, biosynthetic processes, cell cycle, DNA repair, and gene transcription and regulation (Figure 3). On the other hand, the monochromatic subnetworks active in differentiated cells were involved in development and differentiation of various cell types, tissues and organs (Figure 3). We also noted another interesting class of subnetworks that showed mixed changes in expression, including a combination of up and down-regulated genes, whose patterns matched across species. This class may highlight pathways that require or at least exhibit dramatic imbalances in gene expression to maintain stem cell state.

### Comparison to gene expression overlap

We compared conserved subnetworks discovered by our approach to gene sets obtained from a simple intersection of orthologs on the human and mouse differentially expressed gene lists. One might suggest that a reasonable approach to finding the core conserved modules underlying stem cell pluripotency is to simply analyze the most extreme differentially expressed genes in both species. We attempted this approach by comparing the top 600 differentially expressed genes from mouse and human, which is comparable to the total number of genes contained across our subnetworks. There was relatively low overlap between the gene sets: of the 600 genes, only 36 are up-regulated in the both species while 34 are down-regulated (Figure S4). This level of agreement is higher than the number expected by chance (∼15–20), but certainly not as high as one might expect, suggesting that there are a number of core modules that do not exhibit the most extreme expression changes. The overlap does improve when we consider any genes that show significant changes in expression (FDR 5%): 1367 genes are significantly up-regulated in pluripotent stem cells in both human and mouse while 986 are significantly down-regulated, which reflects an overlap of ∼50% (Table 1). However, this more lenient cutoff yields thousands of candidate genes to consider, which makes determination of the core conserved modules difficult. Our conserved subnetworks offer a solution to this problem: we find 255 modules containing approximately 600 genes that appear in both the human and mouse subnetworks, including 282 that are differentially expressed and show similar expression patterns. Simultaneous network discovery guided by the combined differential expression data allows us to directly identify the core conserved patterns of expression, even where some of these patterns are subtle but consistent.

**Table 1:** Gene expression overlap.

	Mouse Genes	Human Genes	Intersection*
Differentially expressed genes	8141	5353	3282
Up-regulated in stem cells	3955	3028	1367
Down-regulated in stem cells	4186	2325	986
Number of genes covered by subnetworks	607	607	601
Subnetwork genes which are up-regulated	214	181	153
Subnetwork genes which are down-regulated	220	214	129

*orthology clusters which belong to both the relevant mouse and human genes.

We were intrigued by the fact that our conserved subnetworks actually contained a significant fraction of genes (∼20%) that showed no evidence of differential expression. By its design (see Materials and Methods, Algorithm), the subnetwork discovery algorithm can include non-differentially expressed genes in identified subnetworks if they connect across highly differentially expressed genes. Briefly, for a given seed gene, the algorithm starts by finding the surrounding functional neighborhood of that seed, which is defined as the set of genes that can be reached within a given path confidence (the product of linkage weights along the path). From this set of genes in the functional neighborhood, the gene that results in the greatest increase in the network activity score is added to the current subnetwork, including any genes required for its connection to the seed. The addition of the corresponding path can potentially bring in non-differentially expressed genes, which may reflect genes that are causally linked to the corresponding subnetwork but whose activity is simply post-transcriptionally regulated [11]. Their activity may be modulated at the protein level which is typical of transduction pathways that control gene expression programs [11]. For example, *TEP1* is not differentially expressed but is found in an active subnetwork with many well-characterized stem cells genes like *POU5F1* (Figure S5A). TEP1 is involved in telomerase activity [28] and has been shown to be regulated by phosphorylation in breast cancer cells [29]. These examples illustrate the advantages of integrating differential expression data with the broader relationships captured by functional linkage networks in that complete modules can be identified, including genes whose activity is not necessarily transcriptionally regulated.

The subnetworks also sometimes contain mixed expression signatures (both up- and down-regulated genes) that are conserved across species, highlighting genes in the same pathway that are antagonistic or genes that exhibit different interactions at various stages of development. For example, one conserved network with mixed expression changes was centered about the important extracellular structural protein ostepontin (also known as secreted phosphoprotein 1, SPP1) (Figure S5B). SPP1 is highly up-regulated in both mouse and human stem cells while its surrounding subnetwork is significantly down-regulated in comparison to differentiated cells in both species. Osteopontin is known to be highly expressed in bone and other cell types like smooth muscle cells, endothelial cells and hematopoietic stem cell niches. The subnetwork captures some well-known interactions of SPP1 in these cells. For example, osteopontin has been shown to be a ligand for CD44 in tumor cells [30]. Pou5f1 has been shown to bind to the preimplantation enhancer element of osteopontin, and thus, the expression of the two proteins is highly correlated in early mouse embryonic development [31]. The induction of osteopontin in immortalized mouse embryonic fibroblasts, in response to TGF-β2, has been shown to promote the maintenance of undifferentiated human embryonic stem cells [32]. This is attributed to the presence of a TGF-β responsive element in the osteopontin enhancer. Thus, osteopontin likely plays a pivotal role in the maintenance of both human and mouse embryonic stem cells, and this subnetwork supports this idea. The functional linkages of osteopontin in early embryonic cells have not been fully elucidated yet, but this subnetwork suggests that this gene may play a role in the embryonic context since the other genes in the subnetwork show an opposing expression pattern. These interesting cases would not be readily discovered through a simple comparison of differential expression lists across species.

### Comparison to other single-species network discovery methods

**Figure 4:**
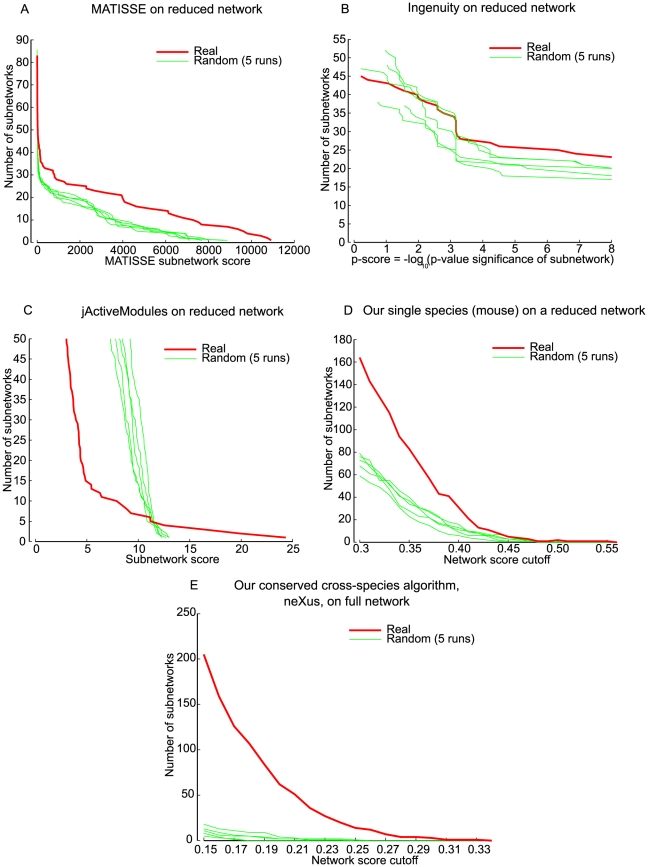
Comparison with other methods. The number of real subnetworks and random subnetworks at various network score cutoffs are plotted for MATISSE (A), Ingenuity (B), jActiveModules (C) and the single-species version of our algorithm (D). The network scores are the metric used by each algorithm to rank the subnetworks. Random subnetworks were obtained by running respective algorithms on the expression data, whose gene labels have been randomly shuffled. Each of the methods uses different forms of the expression data: MATISSE uses expression profiles; jActiveModules uses significance values of the genes; Ingenuity uses focus genes, for which we took any differential expressed gene whose log fold change value was greater (lesser) than 20% of the maximum (minimum) of the most up-regulated (down-regulated) gene; Our method uses fold change scores from the SAM analysis. The scale of the functional linkage network was reduced for all methods shown in (A–D) for a fair comparison. The cross species algorithm on the full network has also been shown for a complete comparison (E).

To our knowledge, our method is the first attempt to interpret differential expression data by integrating with interaction networks across multiple species. Thus, we further assessed the advantages of simultaneous, cross-species network search as compared to active subnetwork discovery in a single species, which has been the focus of previous methods [5], [6], [8], [9], [10], and is the principle behind commonly used analysis tools such as Ingenuity Pathway Analysis (Ingenuity® Systems, www.ingenuity.com). Analogous experiments to those performed on our cross-species algorithm were applied to discover active subnetworks in the mouse functional linkage network alone (see Materials and Methods). Most of the existing approaches did not scale to the complete functional linkage network used by our approach (Table 2), so we reduced the scale of the mouse functional linkage network by restricting the network to the 50,000 highest weight edges to allow for a direct comparison of our approach to other methods in the single-species context. We implemented MATISSE [33], jActiveModules [5] and Ingenuity (Ingenuity® Systems, www.ingenuity.com) on the mouse data and compared with a single-species version of our approach as well as our cross-species algorithm. For methods that do not incorporate weighted edges, we binarized the reduced network. To allow a direct comparison of the number of subnetworks produced by each approach, subnetworks were sorted in descending order by size and overlapping subnetworks were removed when their overlap with larger networks (in genes) was greater than 60%. To estimate the significance of the subnetworks identified by each algorithm, we randomized the gene labels in the expression data and ran each algorithm five times on randomized expression data. The number and scores of subnetworks produced by each algorithm were compared with the number and scores of the subnetworks generated from the 5 runs on randomized expression data (Figure 4).

**Table 2:** Comparison to previous approaches.

First Author	Year	# Nodes	# Edges	Weighted edges	# subnetworks reported in the study	Average size of subnetwork (# nodes)
Ideker [5]	2002	77	362	No	5	11.4
Rajagopalan [6]	2004	9000	30000	No	∼100	34–50
Cabusora [9]	2005	106	233	No	2	65
Ulitsky [33]	2007	6230	89327	No	20	105.35
Guo [7]	2007	6509	23157	No	1	2181
Dittrich [10]	2008	2034	8399	No	1	46
Ulitsky [8]	2009	6220	63989	Yes	14	33.6
Our study - mouse		17868	2700000	Yes	116	11.7
Our study - human		15806	6000000	Yes	127	16.6
Our study - Cross species (neXus)				Yes	255	22

Although our main contribution in this work is the cross-species algorithm, we found a single-species version of our approach performed favorably in comparison to existing approaches (Figure 4). Specifically, it produced more subnetworks than other approaches on the real expression data while producing far fewer subnetworks on the randomized data (Figure 4). Surprisingly, we found that 2 of the 3 existing approaches (Ingenuity and jActiveModules) produced as many or more networks on the randomized data as on the real data for most score cutoffs (Figure 4B–C). Among the existing methods we evaluated, MATISSE provides the best performance, often reporting 1.5–2 fold more real networks at a given score cutoff than on randomized data (Figure 2A). There was significant variation in the size of subnetworks produced across the various approaches, with some producing networks as large as 2000 genes and others producing relatively small subnetworks consisting of less than 10 genes (Figure S6). The most useful number and size of networks will, of course, depend on the application, but one particularly unique feature of our implementation is that subnetworks are captured at all stages of their growth, thus giving the user to control of the tradeoff between size and significance of the subnetwork in consideration (see Web Interface section).

Perhaps the most striking result of our comparison was our finding that any single species approach, including our own, performed much worse than our cross-species subnetwork discovery algorithm. For example, in the single-species setting for mouse, we were able to find 164 subnetworks while discovering an average of 71 (standard deviation of 7.8) subnetworks in our randomization experiments under the same setting (mouse, clustering coefficient threshold = 0.1, network score cutoff = 0.3), suggesting an enrichment of approximately 2.5-fold (Figure 4D). Using the cross-species approach, we found 234 subnetworks while discovering an average of 9.8 (standard deviation of 4.16) in our randomization experiments (parameter setting: mouse and human clustering coefficient thresholds = 0.1 and 0.2, network score cutoff = 0.15), which represents a 20-fold enrichment (Figure 2B). Thus, not only did we discover more candidate networks in the cross-species setting, but the networks we found were of higher statistical confidence. Similar results were obtained when we applied our single-species approach to the complete functional linkage network (Figure S7).

The improvement in sensitivity and specificity by the cross-species approach is a particularly interesting result because it suggests that simultaneous cross-species network discovery can serve as an effective means of improving the signal-to-noise ratio in network discovery even if one is not necessarily interested in asking questions about conservation across species. More pessimistically, this result suggests that separating biologically relevant active subnetworks from random networks based on a single functional linkage network is a challenging problem.

The enhanced performance of the cross-species approach can be attributed to the fact that coordinated expression changes can be reasonably clustered in both species' functional linkage networks. Due to the small-world nature of functional linkage networks (or protein-protein interaction networks) [34], given a large set of genes, subnetworks involving partitions of this set can often be readily found even if these genes do not necessarily play a specific role together. The coherent grouping of genes across species eliminates random aggregation of active genes, and thus, the cross-species approach is able to relax both the network score and clustering coefficient stringency criteria, while still maintaining statistical confidence in the networks. Indeed, when our approach was applied independently to mouse and human data, we found little intersection among the two species' subnetworks: of the genes covered by human (305 orthologous clusters) and mouse subnetworks (261 orthologous clusters), only 21 were overlapping. In contrast, the cross-species approach discovers around 250 subnetworks covering 607 genes in both mouse and human (Table 1). We obtained a similar result when comparing to subnetworks derived from another approach, MATISSE, applied to the human and mouse data (see Text S1, Note 4, “Comparison of the overlap of mouse and human subnetworks discovered through MATISSE and neXus”, Table S1, S2). Thus, in addition to the underlying biological question of conservation of expression signatures, cross-species analysis can serve as an effective noise filter, which is critical for discovering clustered patterns of expression changes in a dense interaction network.

The difficulty in identifying subnetworks from a list of genes within a single species has important implications for how the statistical significance of such networks should be assessed. This problem often arises in practice during the interpretation of candidate gene lists. For example, analysis tools such as Ingenuity Pathway Analysis (Ingenuity® Systems, www.ingenuity.com) are now being widely used based on the single-species discovery method we evaluated above. The significance of networks identified by such approaches are typically assessed by comparing the network score after optimization to scores that obtained by randomly sampling a similarly sized set of genes. However, as demonstrated above, high-scoring networks are often obtained when search algorithms are applied to randomly selected candidate genes. Put simply, in many protein interaction networks, random lists of genes are much easier to connect than one might expect. Our results suggest that significance should instead be estimated by applying the network search process (with the same parameters) to several random candidate genes lists, and evaluating the actual scores in the context of the resulting random score distribution.

### Discussion of specific examples

**Figure 5:**
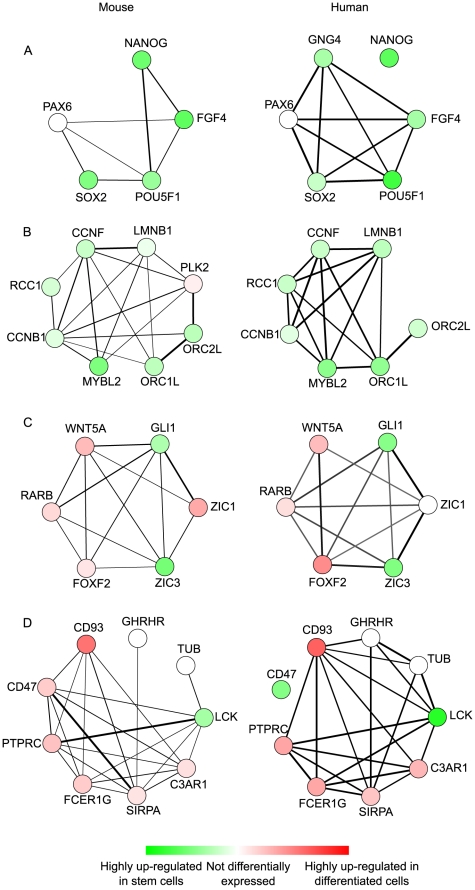
Examples of conserved subnetworks. Subnetworks (A–D) are examples of interesting conserved subnetworks discovered by the cross-species network search algorithm on differentially expressed genes between stem cells and differentiated cells. Each subnetwork represents a subgraph of mouse (left column) and human (right column) functional linkage networks, respectively. Nodes are genes and they are colored green if the gene is up-regulated in stem cells when compared to differentiated cells and red if down-regulated in stem cells relative to differentiated cells. The intensity of green or red color of the genes represents the normalized fold change of the expression. The edge thickness in the subnetworks represents the edge confidence based on the functional linkage networks. The subnetwork (A) shows a conserved subnetwork which contains important stem cell transcription factors. The subnetwork (B) highlights cell cycle related pathway genes. The subnetworks (C, D) are mixed subnetworks, as they contain both up-regulated and down-regulated genes. The genes are functionally related but their mode of function is antagonistic in nature.

Using the cross-species network discovery algorithm, we are able to find subnetworks reflecting conserved functional modules between mouse and human pluripotent stem cells. We found many of these subnetworks to be monochromatically active in stem cells or differentiated cells. This was not a prerequisite for network discovery, but reflects that the majority of genes supporting a local process are regulated in the same direction. Monochromatic subnetworks up-regulated in stem cells were our primary focus because these reflected potential candidate processes that are necessary for maintaining a pluripotent, self-renewing stem cell state. One of the most significant conserved subnetworks of this type captures the core pluripotency circuit in embryonic stem cells (Figure 5A). This network recovers associations between important transcription factors such as *POU5F1*, *NANOG*, *SOX2* and *FGF4*,all of which have been shown to form an important transcriptional circuit in embryonic stem (ES) cells, consisting of feed-forward and autoregulatory loops [35]. Chromatin immunoprecipitation experiments have shown that these three proteins exhibit a significant overlap in their binding sites in the genome [35], [36]. The subnetwork links FGF4 to the core signaling circuitry formed by POU5F1, SOX2, and NANOG. FGF4 has been shown to be expressed in the peri-implantation mouse embryo [37] and the SOX2/POU5F1 complex has been shown to activate transcription of *FGF4* by binding to an enhancer element [38]. The role of this module has also been studied quite extensively in early embryonic development. FGF4 null mutants in mouse are embryonic lethal due to defective primitive endoderm [39]. The cells of the mouse inner cell mass (ICM) show a reciprocal expression pattern of FGF4 (ligand) and FGFR2 (receptor). It has been shown that the FGF4 secreted by the epiblast precursor cells is crucial to the differentiation and maintenance of cells of the trophectoderm and extraembryonic endoderm lineages [40], [41]. Human ESCs show a striking resemblance to mouse epiblast-derived stem cells in terms of morphology and maintenance culture conditions, amongst other characteristics [42], [43]. Thus, this network highlights a core, conserved module active in the pluripotent cells of both the species, irrespective of the downstream effects on cell signaling and morphology. *FGF4* stimulation of ERK1/2 signaling in mouse ES cells has been shown to facilitate lineage commitment [44]. In human ES cells, FGF signaling promotes self-renewal by directly affecting the expression of *NANOG* [45], [46] as well as suppressing expression of genes responsible for reversion to an ICM-like state [47].

Another highly significant subnetwork discovered by our approach pertains to the control of cell cycle progression in ES cells (Figure 5B). Both human and mouse ES cells have a very short G1 phase which can be attributed to the constitutively active CDK2/6 [48], [49]. CCNB1 and MYBL2 are two important cell cycle regulators that are expressed at high levels in undifferentiated ES cells and their expression decreases rapidly upon induction of differentiation [50]. This happens even before loss of the important regulator proteins such as POU5F1 or NANOG can be detected. The conserved subnetwork highlights the role of these two genes in the maintenance of cell cycle progression in ES cells. Knockdown of *MYBL2* has been shown to induce polyploidy/aneuploidy in ES cells and *CCNB1* is a known target of *MYBL2* [51]. *B-MYB* is also crucial for inner cell mass development in mice embryos [52]. The role of *CCNF* in embryonic stem cells has not been explored but yeast two hybrid assays have shown that the NLS domain of CCNF can regulate nuclear localization of CCNB1 [53].

Many conserved subnetworks also included genes that are up-regulated during the initiation of differentiation. This supports the idea that the maintenance of ES cell phenotype requires the suppression of differentiation-associated gene expression as well. One interesting example of this phenomenon was highlighted in a third subnetwork discovered by our approach, which was centered on the protein ZIC3 (Figure 5C). *ZIC3* has been shown to be required for maintaining pluripotency of mouse embryonic stem cells by suppressing endoderm specification [54] while *GLI1* has an important effect on embryonic stem cell proliferation [55]. These two proteins are known to work in coordination for transcriptional activation or repression [56]. Both of these genes code for DNA binding zinc finger proteins and they share and recognize highly conserved zinc finger domains. The down-regulated genes in the subnetwork, namely, *WNT5A*, *FOXF2* and *RARB*, play important roles in the differentiation of embryonic stem cells [57], [58], [59]. It is interesting to observe that these genes have GLI binding sites in their promoter region or cis-regulatory domains, which suggests that GLI1 and ZIC3 could potentially regulate their expression in ES cells [60], [61]. Also, GLI proteins participate in regulation of Hedgehog signaling, of which RARB and FOXF2 are members, and GLI is also known to regulate the members of WNT family [62]. These functional interactions and coordinated expression strongly suggest ZIC3 and GLI1 might be responsible for suppressing the expression of genes such as *FOXF2*, *WNT5A* and *RARB*.

This network in particular provides an illustrative example of how subnetwork discovery can provide novel testable experimental hypotheses. This hypothesis could be explored experimentally through RNAi knockdown of *ZIC3* and *GLI1* in embryonic stem cells to check for resultant changes in expression of the other genes in the network. Lim *et al.* [54] conducted RNAi knockdown of *ZIC3* in human and mouse ESCs and saw enhanced expression of endodermal transcripts like *SOX17* and *PDGFRA*. Further experiments could also be used to check for direct binding of *ZIC3* and *GLI1* to the promoter regions of the differentiation-associated genes. The subnetwork also highlights the striking observation that the gene *ZIC1*, despite sharing 69% homology with *ZIC3*, does not show the same trend in expression in either mouse or human pluripotent stem cells. While *ZIC2* and *ZIC3* have been suggested to have partially overlapping or redundant roles in suppressing endoderm in embryonic stem cells, the role of *ZIC1* in this context has been not been explored much. Further overexpression studies of this gene could be used to elucidate its exact role in this network.

Another interesting subnetwork found by our approach was centered around the seed gene SIRPA. The only gene in the whole subnetwork that is found to be up-regulated in mouse and human pluripotent stem cells is *LCK* (Figure 5D). *LCK* is one of the eight SRC family kinase genes, which are known to play crucial roles in regulating signals from a variety of cell receptors, affecting a variety of cellular processes such as differentiation, growth and cell shape [63]. Members of this family, namely *Hck* and *Lck*, have been implicated in the maintenance of self-renewal of murine embryonic stem cells [46]. *Cyes*, along with *Hck*, have been shown to be regulated by LIF in mouse embryonic stem cells and the expression of their active mutants allows the maintenance of these cells at lower concentrations of LIF [64]. Other studies have also reported the evolutionarily conserved transcriptional co-expression of *LCK* in human and mouse embryonic stem cells based on transcriptomic studies [65]. LCK has also been shown to induce STAT3 phosphorylation and this is believed to cause transformation of cells having constitutive LCK activity [45]. All of the other genes in the sub-network are down-regulated in ES cells, which may be due to the fact that the expression of *SFK*s is generally associated to lineage-restricted patterns in the adult, such as, the expression of *LCK* in T lymphocytes.

While the hypotheses suggested by the discovered subnetworks ultimately require experimental follow-up, these examples illustrate that the networks capture many of the well-characterized processes supporting stem cell pluripotency as well as implicating some novel players. In general, the process of active subnetwork discovery can play an important role in interpreting differential expression or other genome-wide data. Active subnetworks, and in particular those that are conserved across species, provide evidence that a whole process or pathway is up/down-regulated, which is more definitive than the type of information provided by a differential expression list, for example. A single highly differentially expressed gene is less compelling than an entire functional module with evidence of differential expression. Furthermore, because the underlying functional linkage networks are based on large collections of genomic data, our approach can potentially identify functional modules that are not yet characterized, but that play a critical role under the conditions being studied.

### Discovery of species specific subnetworks

**Figure 6:**
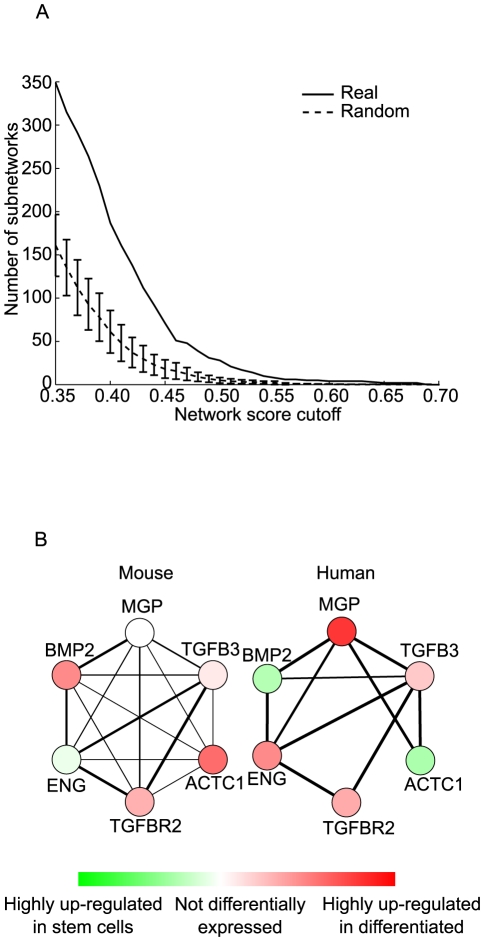
Species specific subnetwork. (A) The number of species-specific subnetworks discovered is plotted versus the network score cutoffs and compared with the number of subnetworks generated by applying the same approach after randomly shuffling gene labels in the expression data. Species-specific networks represent subnetworks with highly divergent patterns across species. (B) An example species-specific subnetwork that highlights the difference in expression of BMP2 pathway related subnetwork in human and mouse. The subnetwork nodes are genes, whose color represent whether are they are active in stem-cells (green) or differentiated cells (red) and intensity of the color represent the degree of expression activity. The thickness of edges of the subnetwork represents the edge confidence based on the functional-linkage network.

We modified the cross-species network discovery algorithm to discover subnetworks that are markedly different in the expression patterns between the two species (see Materials and Methods, “Score of a Subnetwork”). These subnetworks represent tightly interconnected groups of genes or proteins that are active only in one of the species or where the expression changes are in opposite directions, highlighting places where pluripotent stem cell signaling differs between human and mouse. Through randomization experiments similar to the conserved subnetwork identification approach (see Materials and Methods) we found that we were able to find such non-conserved network signatures approximately twice as frequently as on randomized expression profiles (Figure 6A). We note that this is a substantially lower signal-to-noise ratio than for the conserved subnetwork discovery approach, for which we achieved approximately 20-fold improvement over random, suggesting that statistically significant species-specific active subnetworks are harder to discover. This is not surprising given that the relatively frequent appearance of random subnetworks in a single species (Figure 4D), which cannot be easily classified as statistical artifacts or biologically relevant changes across species. The species-specific network discovery problem is not able to take advantage of the noise filtering property of the conserved network search described above.

Nevertheless, we find interesting subnetworks which highlight differences between gene expression in mouse and human stem cells. For example, one species-specific subnetwork (Figure 6B) recapitulates the well-known difference in BMP signaling between human and mouse embryonic stem cells. Mouse embryonic stem cells require BMP2/BMP4 to induce the expression of Inhibitor of differentiation (Id) genes via Smad pathway for self-renewal [66]. Thus, exogenous addition of LIF and BMP4/2 is required to maintain mouse ES cells in culture without differentiation. On the other hand, human ES cells cultured in unconditioned medium exhibit high levels of BMP signaling which causes the cells to differentiate. Mouse epiblast stem cells, like human ES cells, differentiate to trophoectoderm upon BMP4 induction [42]. This needs to be suppressed through an antagonist such as noggin to maintain these cells in an undifferentiated, self-renewing state [46]. The other genes in the subnetwork that show opposite trends in differential expression between human and mouse ES cells are MGP, ACTC1 and ENG. Endoglin (Eng) is an accessory receptor for several TGF-β growth factors, including BMP2, and has been shown to be crucial for embryonic hematopoiesis [67]. Matrix GLA protein (MGP) is a small matrix protein that has been shown to have a direct interaction with BMP2 and has been shown to modulate BMP signaling [68]. The potentially disparate role of these genes in mouse and human ES cells can be explored further.

### Web interface

To facilitate public access to active cross-species subnetworks identified by our approach, we developed a web-based interface for convenient browsing of conserved and species-specific stem cell expression signatures (http://csbio.cs.umn.edu/neXus/subnetworks, download subnetworks in raw text from http://csbio.cs.umn.edu/neXus). Subnetworks are listed according to their corresponding network seed gene, and when a seed gene is selected, the following information is displayed: the conserved active human and mouse subnetworks, significance of the identified subnetwork based on a comparison to network randomization, expression fold changes and name details of mouse and human genes, and the function enrichments of the genes in respective to human and mouse subnetworks based on the Gene Ontology [27]. The subnetwork generation was automated using neato, a Graphviz graph plotting tool with spring model layouts [69]. The Cytoscape version of the subnetworks are also available on the website, which are linked using Cytoscape Webstart [70]. The gene names in the subnetwork are linked to gene information at the Mouse Genome Informatics (MGI) database [71] and GeneCards [72] for mouse and human genes, respectively. Another useful feature of our web-interface is that subnetworks can be interactively expanded based on the cross-species discovery algorithm, which allows for real-time analysis of additional candidate genes that are closely associated with the network of interest. As networks are expanded, a statistical significance score is calculated after each iteration, which allows the user to estimate the potential biological relevance of the network as it is expanded.

### Conclusion

We have described a scalable approach for discovering conserved active subnetworks across species. Starting from candidate gene lists reflecting parallel differential expression studies in two different species, we are able to search for dense subnetworks with conserved patterns of differential expression. In contrast to previous active subnetwork discovery algorithms, our approach not only extends this idea across species, but also enables application of the approach to functional linkage networks as opposed to sparse protein-protein interaction networks. Functional linkage networks integrate information from a diverse collection of genomic and/or proteomic studies (including protein-protein interactions), and thus offer the potential for more sensitive discovery of active subnetworks, including those which involve previously uncharacterized genes.

We applied our approach to a differential expression study between pluripotent mouse and human stem cells versus their differentiated cell types to produce several hundred subnetworks that reflect conserved changes between mouse and human. Network search across species produced specific hypotheses about conserved and differentiated mechanisms of stem cell maintenance, and importantly, demonstrated that such an approach can be an effective means of filtering noise from the active subnetwork discovery problem. We found that identifying statistically significant active subnetworks independently within a single species may be a harder problem than previously appreciated, and we suggest the cross-species approach as one solution to this problem.

Despite the success of our approach, there are a number of promising directions for further improvement and broader application of the method. While the approach was successfully applied to relatively dense functional linkage networks for mouse and human, it is a computationally challenging problem, and the algorithm cannot be applied in real-time as it still requires several days to run. Strategies for improving the efficiency of conserved network discovery and more formal selection criteria for the parameters associated with our approach are both useful future directions. Furthermore, the approach can be readily extended to discover conserved subnetworks across more than just two species, which will make another fruitful direction as we begin to accumulate functional genomic data across a broad variety of other model organisms. Finally, although our study focused on the interpretation of candidate gene lists derived from differential expression analyses, the algorithm is general and can be readily applied to interpret lists arising from other genomic screens, including, for example, genome-wide association studies.

## Materials and Methods

### Microarray data processing

249 mouse microarray data samples were obtained from 20 GEO datasets (Table S3). All the samples had been hybridized to the Affymetrix mouse chip MOE 430 2.0. 132 human microarray data samples were obtained from 12 GEO datasets (Table S4). All the samples had been hybridized to the Affymetrix human chip HGU 133 plus 2.0. The raw data was normalized using the MAS 5.0 algorithm [73] and the average chip intensity was scaled to 500. The probes set IDs with detection p-values higher than 0.4 were termed absent and were filtered out for further analysis, along with the probe set IDs with average intensity lower than 50. Non-negative matrix factorization (NMF) was used to identify major biological classes in the data in both species independently [26]. The algorithm factorizes the expression matrix A into two matrices, W and H. If the expression matrix is of size *N X M*, the algorithm computes an approximation 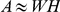, where W and H have sizes *N X k* and *k X M*, respectively [74]. Here, k represents the number of clusters that the samples can be divided into. Each of the k columns of matrix W defines a metagene in such a way that the entry ***w_ij_*** represents the coefficient if gene i in metagene j. Each of the M columns of matrix H depicts the metagene expression profile in different samples such that the entry ***h_ij_*** represents the expression level of metagene i in sample j. The accuracy of the classification is evaluated by the value of the cophonetic coefficient. NMF was used to cluster the samples into biologically meaningful sets. As an example, for k = 6, the mouse samples were clustered into the classes that represented the different levels of pluripotency of the stem cells. The cophonetic coefficient for this classification was 0.978. Similar classification could be achieved for k = 5 in the human gene expression data (cophonetic coefficient of 0.977). As mentioned earlier, the matrix W detected the metagenes representing every cluster of similar samples in the data and, the matrix H gave the expression profile of every sample in the particular metagene. The expression profile of the various samples in the metagene corresponding to the cluster of pluripotent stem cells was used to divide the samples into two major classes, on the basis of the values of the entry ***h_ij_***. Class 1 included the pluripotent ES cells and induced pluripotent stem cells while class 2 represented samples that were in the process of early differentiation or late reprogramming. Differential expression analysis was performed between these two biological classes using Significance Analysis of Microarrays [1]. The results of this differential expression analysis were used as the starting point for subnetwork discovery. The differential expression criteria were set at false discovery rate less than 5%. The results of this differential expression analysis yielded fold changes for significantly differentially expressed genes which was log normalized for both up-regulated and down-regulated genes, separately. The log-ratios were rescaled to ranges from −1 to +1, where −1 represented the gene which is most down-regulated and +1 represented the most up-regulated gene. The majority of the genes were not significantly differentially expressed; the log-ratio of these genes was set to zero. The normalized expression fold change data can be downloaded from http://csbio.cs.umn.edu/neXus.

### Functional linkage networks

We used the mouse functional linkage network previously published in [16] with all edges below 0.10 confidence set to zero, which resulted in around 2.7 million weighted edges among 17868 genes. We obtained the human functional linkage network from [15] (the “global network”) and trimmed the network to the highest 6 million weighted edges, which corresponded to a minimum edge weight of 0.58 and covered 15806 genes.

### Algorithm

The algorithm identifies functional modules enriched for active genes in both species under consideration. Conserved active modules are found based on two criteria: (1) a high degree of clustering in both species' functional linkage networks, and (2) a high average normalized differential expression fold-change (network score) sharing the same sign across species. Because the search space is exponential, a greedy heuristic is applied to expand subnetworks from candidate seed genes. Each candidate network is grown until it fails to meet one of the constraints. This algorithm is implemented in Python and the source code can be downloaded from the supplementary website (http://csbio.cs.umn.edu/neXus) (see Box 1 for pseudocode). Each component of the algorithm is described in more detail below.

**Box 1: Pseudocode for neXus Algorithm**

# assuming global mouseDifferentialGenes, humanDifferentialGenes, mouseFN, humanFN

function subnetworks()

   for seed 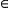 mouseDifferentialGenes 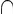 humanDifferentialGenes

   mouseGenesInConsideration = DepthFirstSearch++(seed, mouseFN)

   humanGenesInConsideration = DepthFirstSearch++(seed, humanFN)

   genesInConsideration = mouseGenesInConsideration 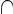 humanGenesInConsideration

   growingSubnetwork = [seed] # list with single gene

       while growingSubnetwork can be grown

          addBestGene(growingSubnetwork, genesInConsideration)

       store subnetwork

   return stored subnetworks

function DepthFirstSearch++(gene, seed, functionalNetwork, threshold)

   for gene 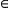 functionalNetwork

      if 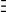 path between gene and seed in the functionalNetwork, such that the product of edge weights in the path exceed threshold, then include the gene. Also store thebest path.

   return included genes

function addBestGene(growingSubnetwork, genesInConsideration)

   return gene in genesInConsideration \ growingSubnetwork such that score(growingSubnetwork+ gene) is the maximum

function score(subnetwork)

   if clustering coefficient of subgraphs of subnetwork in mouseFN and humanFN is not within

   constraints return 0

   return average of score(gene) of all genes in subnetwork

function score(gene) # for neXus, the scoring is simple foldchange[gene] for single species experiment

   return sign(mousefoldchange[gene]*humanfoldchange[gene])* sqrt(abs(mousefoldchange[gene]*humanfoldchange[gene] ))

#### Score of a subnetwork

The network score of a cross-species subnetwork is the average activity scores (described below) of the genes in the two species' subnetworks given that they obey the following constraints: first, the subnetworks satisfy a connectedness constraint on their respective functional linkage network; second, the network score of the subnetwork is above a threshold. In all other cases, the score of the subnetwork is zero. The first condition guarantees that the genes in the subnetwork are interconnected in each species' functional linkage network, which suggests the corresponding set of genes represents a functional module. By enforcing this constraint on both species, conserved modules are selectively chosen. The second constraint guarantees that the subnetwork exhibits a high degree of differential expression, which reflects a coherent response to the phenotype or conditions under consideration.

The connectedness of a subnetwork is quantified by the average weighted clustering coefficient of the subnetwork, which is the ratio of existing connections between the neighbors to the total pairs of neighbors possible. The clustering coefficient for node k is given by 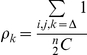, where i, j, k = Δ means nodes i, j, k form a triangle in the graph, and n is total number of neighbors of node k. For a weighted network, the clustering coefficient can be modified to 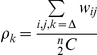 [75], where 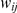 is the weight of the edge ij. Average (weighted) clustering coefficient is the average of the (weighted) clustering coefficients of all the nodes in the graph, which is given by 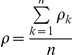. The network score of the subnetwork is the average of the activity scores across all genes in the subnetwork. For single species subnetwork discovery, the normalized fold change of the gene was used as the activity score. For the conserved cross-species approach, the magnitude of the activity scores of genes were calculated as the geometric mean of magnitudes of normalized fold changes of the genes across the two species. The gene activity scores were assigned the same sign as the product of the signs of the normalized fold changes. This means that if the gene was up-regulated or down-regulated in the same direction in both the species, the gene activity score was positive, while genes showing the opposite direction of differential expression were assigned a negative sign. For the species-specific approach, the absolute difference in the normalized fold changes was used as the gene activity score.

#### Growing subnetworks

Subnetworks are grown greedily to optimize the subnetwork score, starting from each gene as a seed. The genes are added from a pool of genes in functional proximity to the seed gene, which are defined by any genes within a minimum path confidence, i.e. the product of all weighted edge confidences in the path, from the seed gene. This pool of genes is discovered using a modification of the depth first search algorithm. Nodes are picked starting from the seed gene, in depth-first fashion, and if the confidence of the path of the searched gene from the seed gene exceeds a threshold (mouse >0.3, human >0.8), it is selected. Subnetworks are grown iteratively by selecting the single gene from the functional neighborhood pool at each stage that maximizes the subnetwork activity score. For each gene in the pool, this score is calculated by adding that gene *in addition to* any genes that are included in its highest confidence path to the current subnetwork. This stage allows interesting non-differentially expressed genes to be added to the subnetwork when they bridge highly differentially expressed genes. Growth of each subnetwork is constrained by two parameters: a minimum network activity score and a minimum clustering coefficient constraint. The first restricts the subnetworks from incorporating too many low-activity genes, while the second ensures that the subnetwork remains highly clustered— genes can only be added if the subnetwork still meets both criteria as described above. Subnetwork growth is stopped when either the clustering coefficient constraint or the minimum network score constraint is not satisfied. This process is repeated for all differentially expressed genes (non-zero activity score).

For the cross-species network discovery approach, the networks are simultaneously grown in parallel. As described above, the activity score is based on the geometric mean of two or more orthologs' normalized differential expression scores, so selected orthologs are added to the respective subnetworks at each step.

#### Orthology

All genes for both human and mouse were mapped to Inparanoid clusters [76]. The clusters contain mapping of genes across species. For human to mouse or vice versa, the majority of ortholog mappings are one to one. However, some of the mappings are many to one, one to many, or many to many. To reduce ambiguity, during comparison, all genes were associated with their corresponding orthologous clusters. The mapping of the functional linkage network from gene space into orthologous cluster space was non-trivial as the interactions of paralogs, genes from the same species in the same orthologous cluster, had to be merged. For a cluster with multiple orthologs, the average of all genes' interactions was assigned as the cluster interaction. For this process, the lack of an edge in the functional linkage network was considered to be a zero weight edge. The outputs of the algorithm, the discovered subnetworks, are reported in the orthologous cluster space.

#### Randomization

To estimate the significance of the obtained subnetworks, randomization experiments were carried out. For both species, the differential expression values were shuffled independently relative to the gene names to remove any connection between them. Fold change values were only shuffled among genes present in the functional linkage network, while the functional linkage network was kept the same. The network discovery algorithm was then run on the shuffled expression data to discover any conserved subnetworks. This entire process was repeated several times to establish a mean and standard deviation for the number of conserved subnetworks identified by chance, which was used to assign confidence values for the real subnetworks. Alternative randomizations schemes provided similar results, and they are described in more detail in Text S1 (Text S1, Note 5, “Other Randomizations”, and Figure S8).

#### Functional coverage of the subnetworks

Gene Ontology [27] enrichment analysis was conducted on each of the subnetworks discovered by our approach using terms from the “biological process” ontology. Significance was assessed using the hypergeometric distribution was used to assess significance [77] and terms with a p-value of less than 0.05 after Bonferroni multiple hypothesis correction were deemed significant. The GO term enrichment analysis results are summarized as a hierarchically clustered matrix with subnetworks as columns and GO terms as rows, where colored elements represent significant enrichment (Figure 3). To distinguish monochromatic subnetworks active in stem cells from the subnetworks active in differentiated cells, we colored the subnetworks green and red, respectively. If the number of genes up-regulated in stem cells is more than twice the number of genes up-regulated in differentiated cells, then the subnetwork is considered active in stem cells and the column corresponding to the subnetwork in the functional matrix is colored green. On the other hand, if the number of genes in the subnetwork up-regulated in differentiated cells is more than twice the number of the genes up-regulated in stem cells, then the subnetwork is active in differentiated cells and is colored red. All the other cases where neither the gene up-regulated in stem cell nor the gene up-regulated in differentiated clearly dominates, the subnetworks are colored yellow in the functional matrix.

## Supporting Information

Figure S1 neXus applied to a single-dataset differential expression analysis. neXus was applied to differential expression lists resulting from analysis of one mouse dataset (GSE3653) and one human dataset (GSE9940). For a clustering coefficient constraint of 0.1 on the mouse network and 0.2 on the human network, we plotted the number of distinct subnetworks generated for a range of network score cutoffs. Overlapping subnetworks were removed when their member genes overlapped more than 60% with larger subnetworks. The number of subnetworks obtained given randomized differential expression values for human and mouse across 5 different random instances is also plotted. We observe a similar enrichment over random subnetworks as in the analysis described in the Results section, demonstrating that the approach applies equally well to smaller-scale differential expression analysis.
(0.03 MB PDF)

Figure S2 Parameter sensitivity analysis to randomized expression data. The cross-species subnetwork discovery algorithm depends on the setting of two parameters: a network score cutoff and a clustering coefficient constraint. Based on 5 random instances in which the differential expression data were shuffled for both species, this figure shows how the number of random conserved subnetworks discovered varies with changes in both the clustering coefficient and network score parameters. This figure can be compared to the parameter sensitivity analysis of real discovered subnetworks (Fig. 2B). All clustering coefficients noted are relative to the background, single-gene average clustering coefficient, which is 0.08 for mouse functional linkage network and 0.35 for human functional linkage network.
(0.03 MB PDF)

Figure S3 Fraction of random to real subnetworks vs. network score cutoff. For a range of network score cutoffs (average normalized fold change), the cross-species subnetwork discovery approach was run on the real differential expression values as well as on several random instances, where the differential expression data were shuffled with respect to the gene labels. At each parameter setting, the ratio of the number of subnetworks obtained from the random instances was measured relative to the number of real subnetworks (noise to signal ratio). The parameters used for this experiment are clustering coefficient 0.1 and 0.2 for mouse and human respectively and >0.15 for network score cutoff.
(0.03 MB PDF)

Figure S4 Analysis of ortholog overlap in differential expression lists vs. conserved subnetworks. To address the question of whether the core conserved modules involved in stem cell pluripotency could be identified by simply comparing the most highly differentially expressed genes in both species, we compared among differentially expressed genes to that obtained from our subnetworks. Specifically, we selected a subset of the significantly differentially expressed genes (based on SAM) that was similar in size to the total number of genes that appear in the human and mouse subnetworks produced by our approach (∼600 genes). This gene list contained roughly half up- and half down-regulated genes. We then measured the intersection (based on our orthology mapping) between the human and mouse gene lists, which resulted in 36 up-regulated and 34 down-regulated genes in common. Although this overlap is highly statistically significant, it is much lower than the overlap between the mouse and human gene lists in the subnetworks produced by our approach (overlap of 601 as compared to 70). The subnetworks from our approach were obtained with clustering coefficient constraints of 0.1 on the mouse network and 0.2 on the human network and a network score cutoff of 0.15.
(0.04 MB PDF)

Figure S5 Example conserved active subnetworks. Subnetworks (a–b) are interesting subnetworks discovered by the cross-species network search algorithm on differentially expressed genes between stem cells and differentiated cells. Each subnetwork represents a subgraph of the mouse (left column) and human (right column) functional linkage networks. Nodes are genes, and they are colored green if they are up-regulated in stem cells relative to differentiated cells. The intensity of the green or red color of the genes represents the normalized fold change in expression. The edge thicknesses in the subnetworks represent the edge confidence based on the functional linkage networks. The subnetwork (a) shows that TEP1 is not differentially regulated in the subnetwork enriched for transcription factor genes. The subnetwork (b) is an interesting case where both up-regulated and down-regulated genes are found in the subnetwork.
(0.04 MB PDF)

Figure S6 Cumulative size distribution of subnetworks generated by existing methods. All methods were run on the mouse reduced functional linkage networks (50,000 highest weight edges). For each method, the subnetworks were sorted in term of the sizes and the sizes were plotted against their rank in the sorted list. The greater the difference between the real and random curve, the greater the confidence we can have in the biological significance of the real subnetworks. To display the utility of our cross species approach, we ran the approach (clustering coefficient parameters >0.1 and >0.2 for mouse and human, respectively and network score >0.15) on the full functional linkage networks which is also shown for comparison.
(0.04 MB PDF)

Figure S7 Evaluation of single species approach. The figures show the comparison of number of real subnetworks to average of random subnetworks over multiple experiments (5), when the single species variant of the network search algorithm was applied to the human and mouse expression data and functional linkage networks. The number of subnetworks identified at increasingly network score criteria is indicated when the algorithm was applied independently to (A) mouse (clustering coefficient criterion >0.2) and (B) human (clustering coefficient criterion >0.5).
(0.03 MB PDF)

Figure S8 Subnetwork evaluation based on alternative randomization schemes. In addition to the randomization scheme described in the Results section, which involves shuffling the differential expression values in both species, we evaluated three other schemes as well: randomizing differential expression values in only mouse, randomizing differential expression values in only human, and randomizing the orthology links between mouse and human. The figure plots the average number of subnetworks discovered across 5 random instances for each scheme with the dotted line providing a reference corresponding to 10% of the subnetworks identified on the real data. At the same parameters at which we discover 255 real subnetworks (clustering coefficient parameters >0.1 and >0.2 for mouse and human, respectively and network score >0.15), we found an average of ∼11 with our original randomization approach, an average of ∼30 with the mouse-only randomization, an average of ∼24 with the human-only randomization, and an average of ∼3 with the orthology randomization. Even by the most conservative randomization scheme, our approach finds ∼10-fold more real networks than random.
(0.03 MB PDF)

Table S1 Overlap between human and mouse genes covered by MATISSE and our cross species algorithm.
(0.17 MB PDF)

Table S2 Analysis of considerable overlap between the subnetworks of the two species obtained through MATISSE and our cross species algorithm.
(0.18 MB PDF)

Table S3 Summary of Mus musculus microarray data.
(0.27 MB PDF)

Table S4 Summary of Homo sapiens microarray data.
(0.27 MB PDF)

Table S5 List of GO Terms enrichments for stem cells, differentiated cells and mixed subnetworks.
(0.19 MB XLS)

Text S1 This document contains the following supplementary notes: Note 1: Implications of using functional linkage vs. physical interaction networks for active subnetwork discovery; Note 2: neXus applied to single dataset differential expression study; Note 3: Independence of the datasets; Note 4: Comparison of the overlap of mouse and human subnetworks discovered through MATISSE and neXus; Note 5: Other randomizations.
(0.42 MB PDF)
