# Supplementary material for: A Scalable Approach for Discovering Conserved Active Subnetworks across Species
Source: PLoS Comput Biol. 2010 Dec 9;6(12):e1001028. doi: 10.1371/journal.pcbi.1001028 (PMC3000367; doi:10.1371/journal.pcbi.1001028)
Supplement: Figure S1 — neXus applied to a single-dataset differential expression analysis. neXus was applied to differential expression lists resulting from analysis of one mouse dataset (GSE3653) and one human dataset (GSE9940). For a clustering coefficient constraint of 0.1 on the mouse network and 0.2 on the human network, we plotted the number of distinct subnetworks generated for a range of network score cutoffs. Overlapping subnetworks were removed when their member genes overlapped more than 60% with larger subnetworks. The number of subnetworks obtained given randomized differential expression values for human and mouse across 5 different random instances is also plotted. We observe a similar enrichment over random subnetworks as in the analysis described in the Results section, demonstrating that the approach applies equally well to smaller-scale differential expression analysis. (0.03 MB PDF) [file pcbi.1001028.s001.pdf]

Figure S1

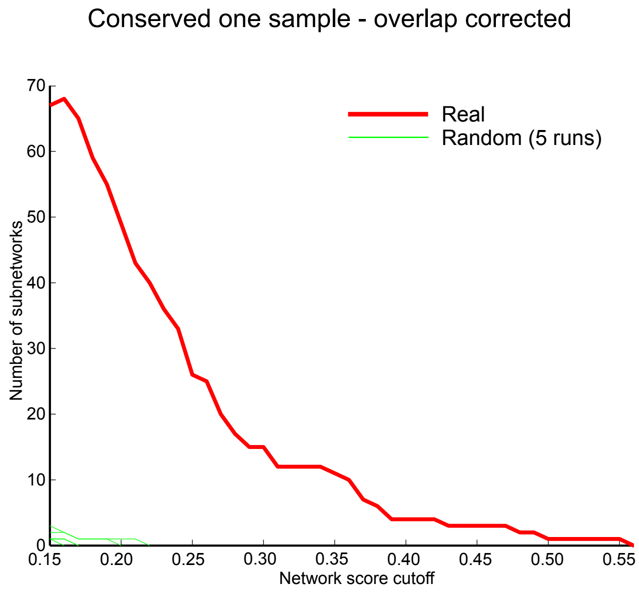

neXus applied to a single-dataset differential expression analysis. neXus was applied to differential expression lists resulting from analysis of one mouse dataset (GSE3653) and one human dataset (GSE9940). For a clustering coefficient constraint of 0.1 on the mouse network and 0.2 on the human network, we plotted the number of distinct subnetworks generated for a range of network score cutoffs. Overlapping subnetworks were removed when their member genes overlapped more than 60% with larger subnetworks. The number of subnetworks obtained given randomized differential expression values for human and mouse across 5 different random instances is also plotted. We observe a similar enrichment over random subnetworks as in the analysis described in the Results section, demonstrating that the approach applies equally well to smaller-scale differential expression analysis.
