# Supplementary material for: A Scalable Approach for Discovering Conserved Active Subnetworks across Species
Source: PLoS Comput Biol. 2010 Dec 9;6(12):e1001028. doi: 10.1371/journal.pcbi.1001028 (PMC3000367; doi:10.1371/journal.pcbi.1001028)
Supplement: Figure S2 — Parameter sensitivity analysis to randomized expression data. The cross-species subnetwork discovery algorithm depends on the setting of two parameters: a network score cutoff and a clustering coefficient constraint. Based on 5 random instances in which the differential expression data were shuffled for both species, this figure shows how the number of random conserved subnetworks discovered varies with changes in both the clustering coefficient and network score parameters. This figure can be compared to the parameter sensitivity analysis of real discovered subnetworks (Fig. 2B). All clustering coefficients noted are relative to the background, single-gene average clustering coefficient, which is 0.08 for mouse functional linkage network and 0.35 for human functional linkage network. (0.03 MB PDF) [file pcbi.1001028.s002.pdf]

Figure S2

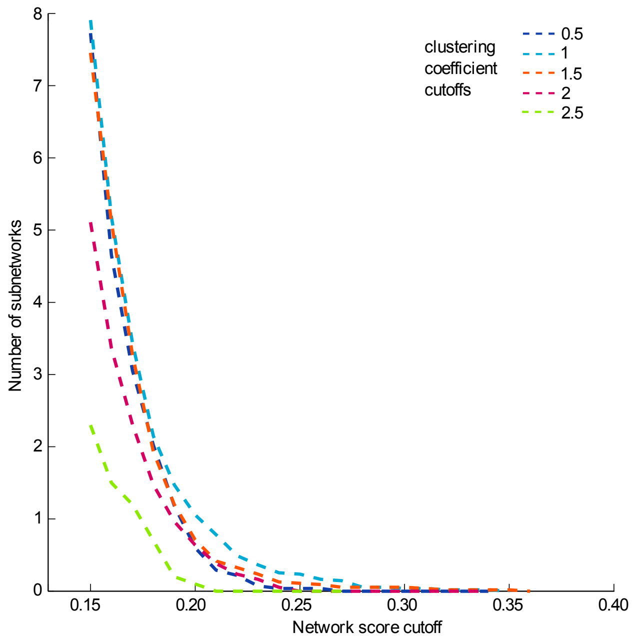

Parameter sensitivity analysis to randomized expression data. The cross-species subnetwork discovery algorithm depends on the setting of two parameters: a network score cutoff and a clustering coefficient constraint. Based on 5 random instances in which the differential expression data were shuffled for both species, this figure shows how the number of random conserved subnetworks discovered varies with changes in both the clustering coefficient and network score parameters. This figure can be compared to the parameter sensitivity analysis of real discovered subnetworks (Fig. 2B). All clustering coefficients noted are relative to the background, single-gene average clustering coefficient, which is 0.08 for mouse functional linkage network and 0.35 for human functional linkage network.
