# Supplementary material for: A Scalable Approach for Discovering Conserved Active Subnetworks across Species
Source: PLoS Comput Biol. 2010 Dec 9;6(12):e1001028. doi: 10.1371/journal.pcbi.1001028 (PMC3000367; doi:10.1371/journal.pcbi.1001028)
Supplement: Figure S3 — Fraction of random to real subnetworks vs. network score cutoff. For a range of network score cutoffs (average normalized fold change), the cross-species subnetwork discovery approach was run on the real differential expression values as well as on several random instances, where the differential expression data were shuffled with respect to the gene labels. At each parameter setting, the ratio of the number of subnetworks obtained from the random instances was measured relative to the number of real subnetworks (noise to signal ratio). The parameters used for this experiment are clustering coefficient 0.1 and 0.2 for mouse and human respectively and >0.15 for network score cutoff. (0.03 MB PDF) [file pcbi.1001028.s003.pdf]

Figure S3

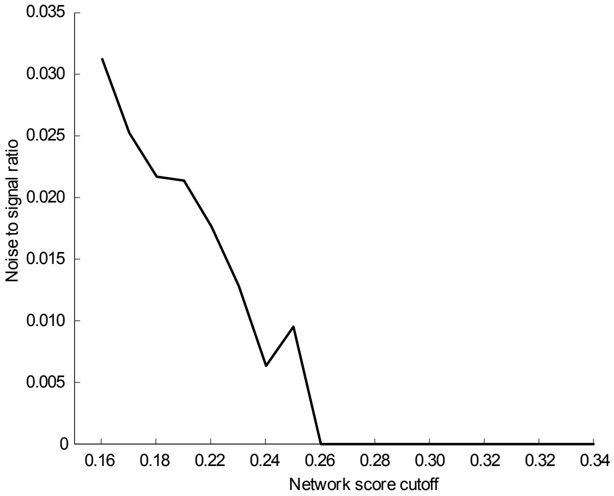

Fraction of random to real subnetworks vs. network score cutoff. For a range of network score cutoffs (average normalized fold change), the cross-species subnetwork discovery approach was run on the real differential expression values as well as on several random instances, where the differential expression data were shuffled with respect to the gene labels. At each parameter setting, the ratio of the number of subnetworks obtained from the random instances was measured relative to the number of real subnetworks (noise to signal ratio). The parameters used for this experiment are clustering coefficient 0.1 and 0.2 for mouse and human respectively and > 0.15 for network score cutoff.
