# Supplementary material for: A Scalable Approach for Discovering Conserved Active Subnetworks across Species
Source: PLoS Comput Biol. 2010 Dec 9;6(12):e1001028. doi: 10.1371/journal.pcbi.1001028 (PMC3000367; doi:10.1371/journal.pcbi.1001028)
Supplement: Figure S4 — Analysis of ortholog overlap in differential expression lists vs. conserved subnetworks. To address the question of whether the core conserved modules involved in stem cell pluripotency could be identified by simply comparing the most highly differentially expressed genes in both species, we compared among differentially expressed genes to that obtained from our subnetworks. Specifically, we selected a subset of the significantly differentially expressed genes (based on SAM) that was similar in size to the total number of genes that appear in the human and mouse subnetworks produced by our approach (∼600 genes). This gene list contained roughly half up- and half down-regulated genes. We then measured the intersection (based on our orthology mapping) between the human and mouse gene lists, which resulted in 36 up-regulated and 34 down-regulated genes in common. Although this overlap is highly statistically significant, it is much lower than the overlap between the mouse and human gene lists in the subnetworks produced by our approach (overlap of 601 as compared to 70). The subnetworks from our approach were obtained with clustering coefficient constraints of 0.1 on the mouse network and 0.2 on the human network and a network score cutoff of 0.15. (0.04 MB PDF) [file pcbi.1001028.s004.pdf]

Figure S4

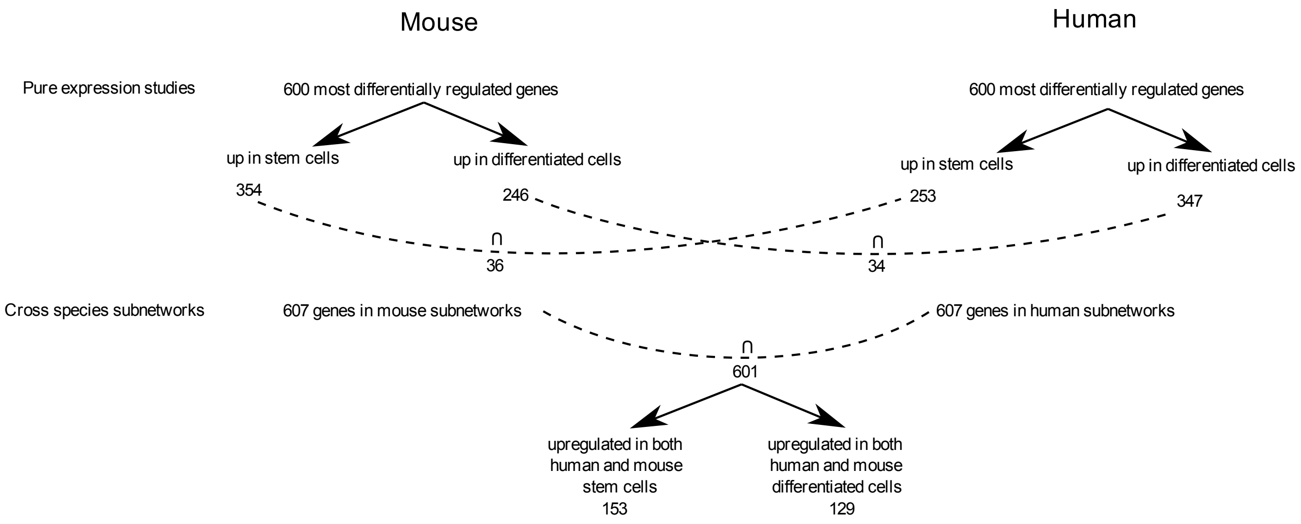

Analysis of ortholog overlap in differential expression lists vs. conserved subnetworks. To address the question of whether the core conserved modules involved in stem cell pluripotency could be identified by simply comparing the most highly differentially expressed genes in both species, we compared among differentially expressed genes to that obtained from our subnetworks. Specifically, we selected a subset of the significantly differentially expressed genes (based on SAM) that was similar in size to the total number of genes that appear in the human and mouse subnetworks produced by our approach (~ 600 genes). This gene list contained roughly half up- and half down-regulated genes. We then measured the intersection (based on our orthology mapping) between the human and mouse gene lists, which resulted in 36 up-regulated and 34 down-regulated genes in common. Although this overlap is highly statistically significant, it is much lower than the overlap between the mouse and human gene lists in the subnetworks produced by our approach (overlap of 601 as compared to 70). The subnetworks from our approach were obtained with clustering coefficient constraints of 0.1 on the mouse network and 0.2 on the human network and a network score cutoff of 0.15.
