# Supplementary material for: A Scalable Approach for Discovering Conserved Active Subnetworks across Species
Source: PLoS Comput Biol. 2010 Dec 9;6(12):e1001028. doi: 10.1371/journal.pcbi.1001028 (PMC3000367; doi:10.1371/journal.pcbi.1001028)
Supplement: Figure S5 — Example conserved active subnetworks. Subnetworks (a–b) are interesting subnetworks discovered by the cross-species network search algorithm on differentially expressed genes between stem cells and differentiated cells. Each subnetwork represents a subgraph of the mouse (left column) and human (right column) functional linkage networks. Nodes are genes, and they are colored green if they are up-regulated in stem cells relative to differentiated cells. The intensity of the green or red color of the genes represents the normalized fold change in expression. The edge thicknesses in the subnetworks represent the edge confidence based on the functional linkage networks. The subnetwork (a) shows that TEP1 is not differentially regulated in the subnetwork enriched for transcription factor genes. The subnetwork (b) is an interesting case where both up-regulated and down-regulated genes are found in the subnetwork. (0.04 MB PDF) [file pcbi.1001028.s005.pdf]

Figure S2

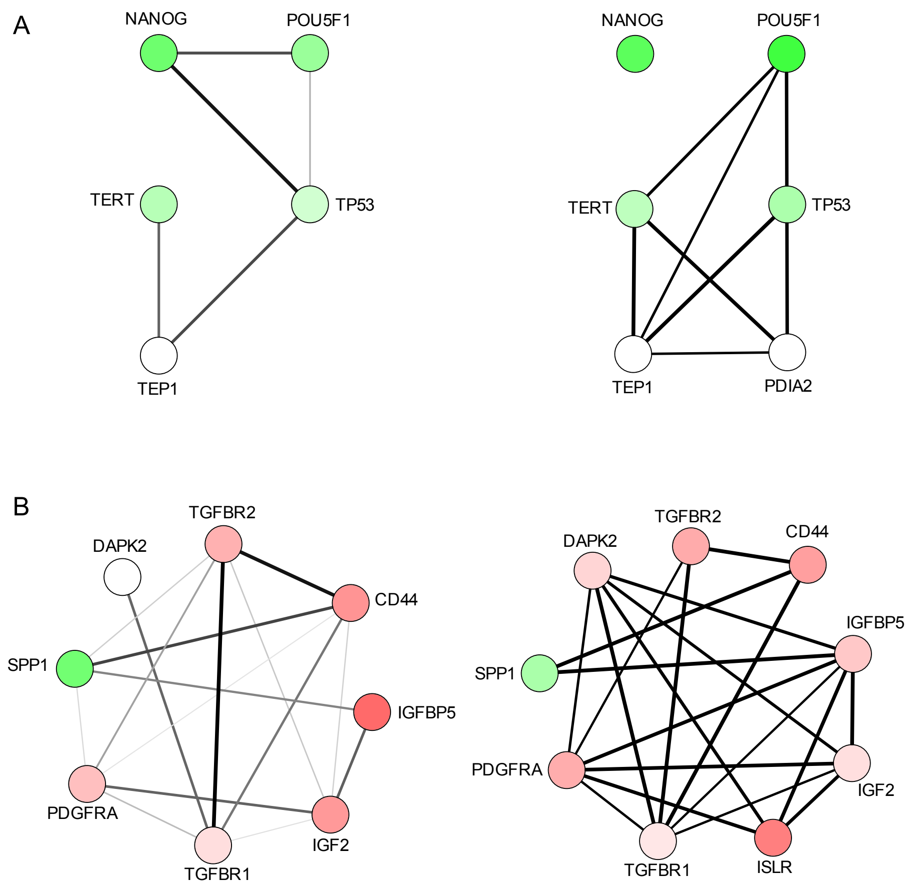

Example conserved active subnetworks. Subnetworks (a-b) are interesting subnetworks discovered by the cross-species network search algorithm on differentially expressed genes between stem cells and differentiated cells. Each subnetwork represents a subgraph of the mouse (left column) and human (right column) functional linkage networks. Nodes are genes, and they are colored green if they are up-regulated in stem cells relative to differentiated cells. The intensity of the green or red color of the genes represents the normalized fold change in expression. The edge thicknesses in the subnetworks represent the edge confidence based on the functional linkage networks. The subnetwork (a) shows that TEP1 is not differentially regulated in the subnetwork enriched for transcription factor genes. The subnetwork (b) is an interesting case where both up-regulated and down-regulated genes are found in the subnetwork.
