# Supplementary material for: A Scalable Approach for Discovering Conserved Active Subnetworks across Species
Source: PLoS Comput Biol. 2010 Dec 9;6(12):e1001028. doi: 10.1371/journal.pcbi.1001028 (PMC3000367; doi:10.1371/journal.pcbi.1001028)
Supplement: Figure S6 — Cumulative size distribution of subnetworks generated by existing methods. All methods were run on the mouse reduced functional linkage networks (50,000 highest weight edges). For each method, the subnetworks were sorted in term of the sizes and the sizes were plotted against their rank in the sorted list. The greater the difference between the real and random curve, the greater the confidence we can have in the biological significance of the real subnetworks. To display the utility of our cross species approach, we ran the approach (clustering coefficient parameters >0.1 and >0.2 for mouse and human, respectively and network score >0.15) on the full functional linkage networks which is also shown for comparison. (0.04 MB PDF) [file pcbi.1001028.s006.pdf]

Figure S6

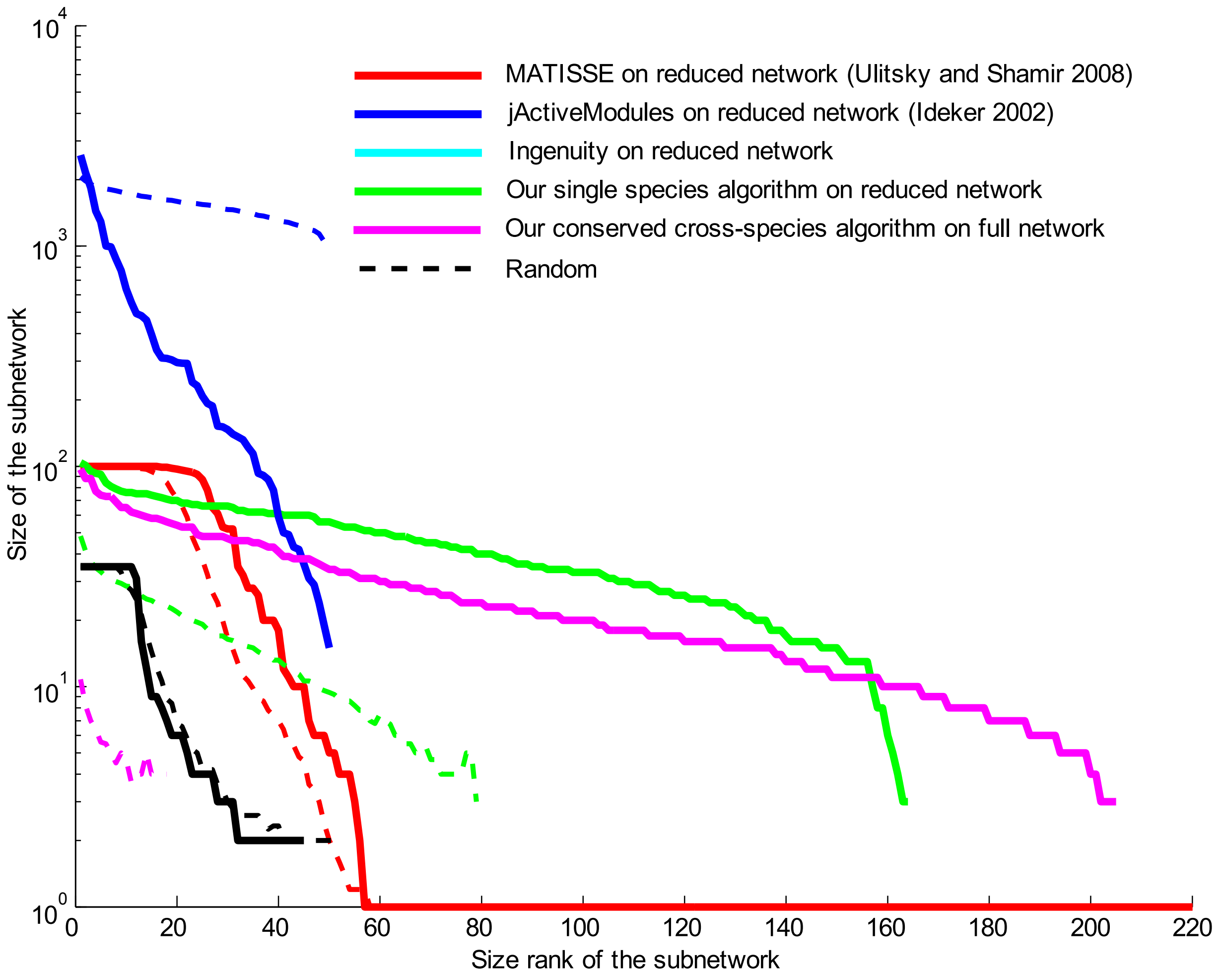

Cumulative size distribution of subnetworks generated by existing methods. All methods were run on the mouse reduced functional linkage networks (50,000 highest weight edges). For each method, the subnetworks were sorted in term of the sizes and the sizes were plotted against their rank in the sorted list. The greater the difference between the real and random curve, the greater the confidence we can have in the biological significance of the real subnetworks. To display the utility of our cross species approach, we ran the approach (clustering coefficient parameters > 0.1 and > 0.2 for mouse and human, respectively and network score > 0.15) on the full functional linkage networks which is also shown for comparison.
