# Supplementary material for: A Scalable Approach for Discovering Conserved Active Subnetworks across Species
Source: PLoS Comput Biol. 2010 Dec 9;6(12):e1001028. doi: 10.1371/journal.pcbi.1001028 (PMC3000367; doi:10.1371/journal.pcbi.1001028)
Supplement: Figure S7 — Evaluation of single species approach. The figures show the comparison of number of real subnetworks to average of random subnetworks over multiple experiments (5), when the single species variant of the network search algorithm was applied to the human and mouse expression data and functional linkage networks. The number of subnetworks identified at increasingly network score criteria is indicated when the algorithm was applied independently to (A) mouse (clustering coefficient criterion >0.2) and (B) human (clustering coefficient criterion >0.5). (0.03 MB PDF) [file pcbi.1001028.s007.pdf]

Figure S7

A

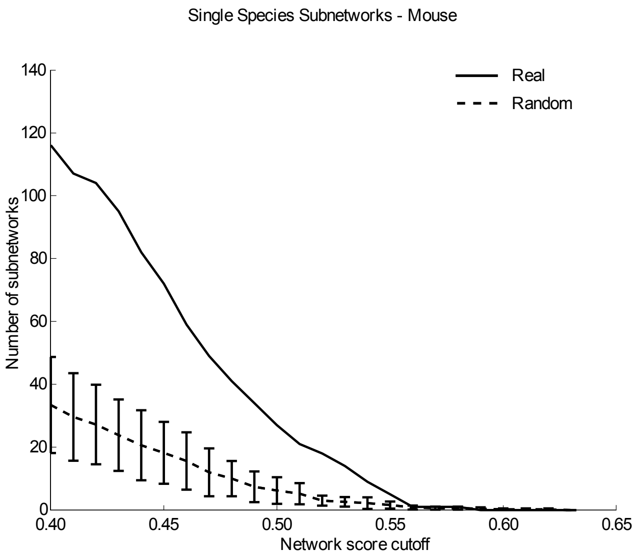

B

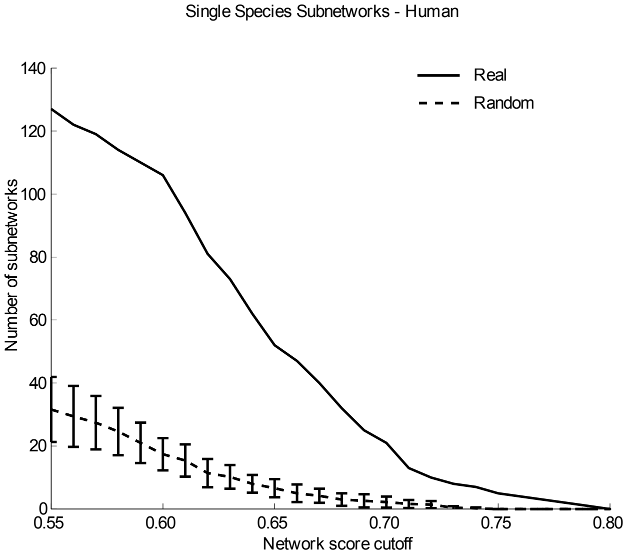

Evaluation of single species approach. The figures show the comparison of number of real subnetworks to average of random subnetworks over multiple experiments (5), when the single species variant of the network search algorithm was applied to the human and mouse expression data and functional linkage networks. The number of subnetworks identified at increasingly network score criteria is indicated when the algorithm was applied independentlyto (A) mouse (clustering coefficient criterion > 0.2) and (B) human (clustering coefficient criterion > 0.5).
