# Supplementary material for: A Scalable Approach for Discovering Conserved Active Subnetworks across Species
Source: PLoS Comput Biol. 2010 Dec 9;6(12):e1001028. doi: 10.1371/journal.pcbi.1001028 (PMC3000367; doi:10.1371/journal.pcbi.1001028)
Supplement: Figure S8 — Subnetwork evaluation based on alternative randomization schemes. In addition to the randomization scheme described in the Results section, which involves shuffling the differential expression values in both species, we evaluated three other schemes as well: randomizing differential expression values in only mouse, randomizing differential expression values in only human, and randomizing the orthology links between mouse and human. The figure plots the average number of subnetworks discovered across 5 random instances for each scheme with the dotted line providing a reference corresponding to 10% of the subnetworks identified on the real data. At the same parameters at which we discover 255 real subnetworks (clustering coefficient parameters >0.1 and >0.2 for mouse and human, respectively and network score >0.15), we found an average of ∼11 with our original randomization approach, an average of ∼30 with the mouse-only randomization, an average of ∼24 with the human-only randomization, and an average of ∼3 with the orthology randomization. Even by the most conservative randomization scheme, our approach finds ∼10-fold more real networks than random. (0.03 MB PDF) [file pcbi.1001028.s008.pdf]

Figure S8

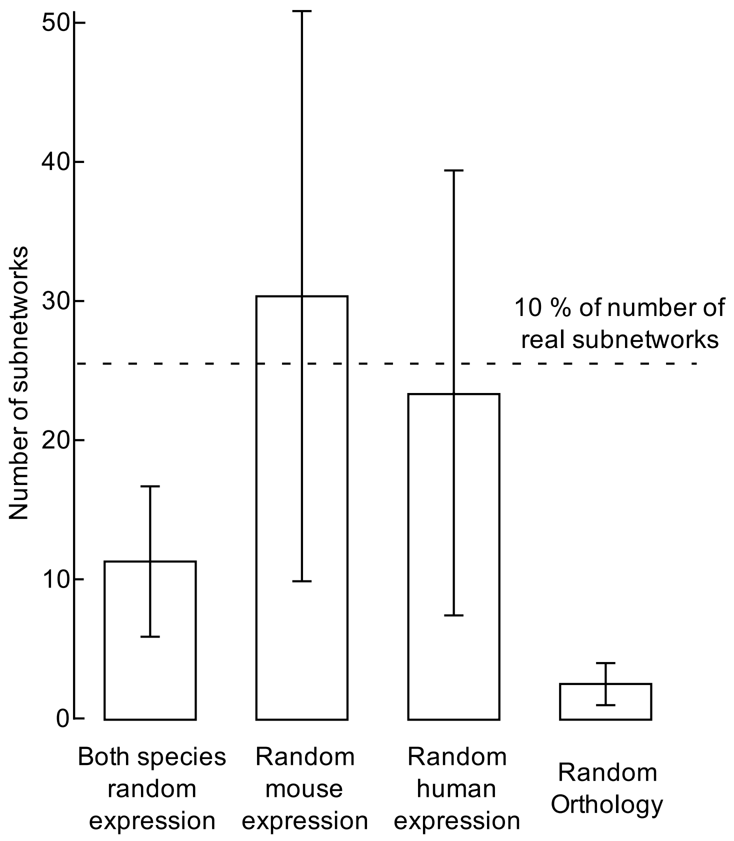

Subnetwork evaluation based on alternative randomization schemes. In addition to the randomization scheme described in the Results section, which involves shuffling the differential expression values in both species, we evaluated three other schemes as well: randomizing differential expression values in only mouse, randomizing differential expression values in only human, and randomizing the orthology links between mouse and human. The figure plots the average number of subnetworks discovered across 5 random instances for each scheme with the dotted line providing a reference corresponding to 10% of the subnetworks identified on the real data. At the same parameters at which we discover 255 real subnetworks (clustering coefficient parameters > 0.1 and > 0.2 for mouse and human, respectively and network score > 0.15), we found an average of ~11 with our original randomization approach, an average of ~30 with the mouse-only randomization, an average of ~24 with the human-only randomization, and an average of ~3 with the orthology randomization. Even by the most conservative randomization scheme, our approach finds ~10-fold more real networks than random.
