# Supplementary material for: A Scalable Approach for Discovering Conserved Active Subnetworks across Species
Source: PLoS Comput Biol. 2010 Dec 9;6(12):e1001028. doi: 10.1371/journal.pcbi.1001028 (PMC3000367; doi:10.1371/journal.pcbi.1001028)
Supplement: Table S1 — Overlap between human and mouse genes covered by MATISSE and our cross species algorithm. (0.17 MB PDF) [file pcbi.1001028.s009.pdf]

Supplementary Table S1. Overlap between human and mouse genes covered by MATISSE and our cross species algorithm

	# Mouse genes	# Human genes	Overlap
**MATISSE**	3256	3773	1619
**neXus**	607	607	601
