# Supplementary material for: A Scalable Approach for Discovering Conserved Active Subnetworks across Species
Source: PLoS Comput Biol. 2010 Dec 9;6(12):e1001028. doi: 10.1371/journal.pcbi.1001028 (PMC3000367; doi:10.1371/journal.pcbi.1001028)
Supplement: Table S2 — Analysis of considerable overlap between the subnetworks of the two species obtained through MATISSE and our cross species algorithm. (0.18 MB PDF) [file pcbi.1001028.s010.pdf]

Supplementary Table S2. Analysis of considerable overlap between the subnetworks of the two species obtained through MATISSE and our cross species algorithm

	**# Mouse subnetworks**	**# Human subnetworks**	**Overlap (Jaccard index > 0.2)**
**MATISSE**	70	92	1
**Our cross species**	255	255	255
