# Supplementary material for: A Scalable Approach for Discovering Conserved Active Subnetworks across Species
Source: PLoS Comput Biol. 2010 Dec 9;6(12):e1001028. doi: 10.1371/journal.pcbi.1001028 (PMC3000367; doi:10.1371/journal.pcbi.1001028)
Supplement: Table S3 — Summary of Mus musculus microarray data. (0.27 MB PDF) [file pcbi.1001028.s011.pdf]

Supplementary Table S3. Summary of *Mus musculus* microarray data

Set	Accession Number	Description	Number of Samples	Reference
1	GSE3653	Differentiation of mouse ESCs by DOX-induction of Ngn3	16	[1]
2	GSE4189	siRNA knockdown of Pou5f1 and Nanog in mouse ESCs	14	[2]
3	GSE13235	mESCs grown in low concentration of Nitric oxide	9	-
4	GSE10210	Endothelial differentiation of mESCs	16	[3]
5	GSE10806	Reprogramming mouse neural stem cells	11	[4]
6	GSE9563	Differentiation of mESCs into embryoid bodies	30	[5]
7	GSE11044	Differentiation of hematopoietic stem cells	6	[6]
8	GSE10574	Eed knockdown in mESCs	2	[7]
9	GSE10477	Conditional Pou5f1 Knockout in mESCs	3	[7]
10	GSE10476	Ring1A\B double Knockout in mESCs	4	[7]
11	GSE10534	Gata6 overexpression in mESCs	3	[7]
12	GSE7528	CYP26A1 Knockout mESCs	18	[8]
13	GSE8503	Dicer deficient mESCs	6	[9]
14	GSE9760	Expanded CAG repeats augment differentiation of mESCs	12	[10]
15	GSE6933	Unique Signature of Multipotent Adult Progenitor Cells	15	[11]
16	GSE5671	mESCs-derived cardiac precusor cells	18	[12]
17	GSE8128	Modeling insertinal mutagenesis in mESCs	9	[13]
18	GSE7688	Genome wide mapping of active promoters in mESCs	5	[14]
19	GSE10871	Dissecting reprogramming through genomic analysis	32	[15]
20	GSE4307	Single cell expression data from mouse ICM	20	[16]

1. Treff NR, Vincent RK, Budde ML, Browning VL, Magliocca JF, et al. (2006) Differentiation of embryonic stem cells conditionally expressing neurogenin 3. Stem Cells 24: 2529-2537.
2. Loh YH, Wu Q, Chew JL, Vega VB, Zhang W, et al. (2006) The Oct4 and Nanog transcription network regulates pluripotency in mouse embryonic stem cells. Nat Genet 38: 431-440.
3. Nikolova-Krstevski V, Bhasin M, Otu HH, Libermann T, Oettgen P (2008) Gene expression analysis of embryonic stem cells expressing VE-cadherin (CD144) during endothelial differentiation. BMC Genomics 9: 240.
4. Kim JB, Zaehres H, Wu G, Gentile L, Ko K, et al. (2008) Pluripotent stem cells induced from adult neural stem cells by reprogramming with two factors. Nature 454: 646-650.
5. Sampath P, Pritchard DK, Pabon L, Reinecke H, Schwartz SM, et al. (2008) A hierarchical network controls protein translation during murine embryonic stem cell self-renewal and differentiation. Cell Stem Cell 2: 448-460.
6. Orford K, Kharchenko P, Lai W, Dao MC, Worhunsky DJ, et al. (2008) Differential H3K4 methylation identifies developmentally poised hematopoietic genes. Dev Cell 14: 798-809.
7. Endoh M, Endo TA, Endoh T, Fujimura Y, Ohara O, et al. (2008) Polycomb group proteins Ring1A/B are functionally linked to the core transcriptional regulatory circuitry to maintain ES cell identity. Development 135: 1513-1524.
8. Langton S, Gudas LJ (2008) CYP26A1 knockout embryonic stem cells exhibit reduced differentiation and growth arrest in response to retinoic acid. Dev Biol 315: 331-354.
9. Sinkkonen L, Hugenschmidt T, Berninger P, Gaidatzis D, Mohn F, et al. (2008) MicroRNAs control de novo DNA methylation through regulation of transcriptional repressors in mouse embryonic stem cells. Nat Struct Mol Biol 15: 259-267.
10. Lorincz MT, Zawistowski VA (2009) Expanded CAG repeats in the murine Huntington's disease gene increases neuronal differentiation of embryonic and neural stem cells. Mol Cell Neurosci 40: 1-13.
11. Ulloa-Montoya F, Kidder BL, Pauwelyn KA, Chase LG, Luttun A, et al. (2007) Comparative transcriptome analysis of embryonic and adult stem cells with extended and limited differentiation capacity. Genome Biol 8: R163.
12. Christoforou N, Miller RA, Hill CM, Jie CC, McCallion AS, et al. (2008) Mouse ES cell-derived cardiac precursor cells are multipotent and facilitate identification of novel cardiac genes. J Clin Invest 118: 894-903.
13. Nord AS, Vranizan K, Tingley W, Zambon AC, Hanspers K, et al. (2007) Modeling insertional mutagenesis using gene length and expression in murine embryonic stem cells. PLoS One 2: e617.
14. Barrera LO, Li Z, Smith AD, Arden KC, Cavenee WK, et al. (2008) Genome-wide mapping and analysis of active promoters in mouse embryonic stem cells and adult organs. Genome Res 18: 46-59.
15. Mikkelsen TS, Hanna J, Zhang X, Ku M, Wernig M, et al. (2008) Dissecting direct reprogramming through integrative genomic analysis. Nature 454: 49-55.
16. Kurimoto K, Yabuta Y, Ohinata Y, Ono Y, Uno KD, et al. (2006) An improved single-cell cDNA amplification method for efficient high-density oligonucleotide microarray analysis. Nucleic Acids Res 34: e42.
