# Supplementary material for: A Scalable Approach for Discovering Conserved Active Subnetworks across Species
Source: PLoS Comput Biol. 2010 Dec 9;6(12):e1001028. doi: 10.1371/journal.pcbi.1001028 (PMC3000367; doi:10.1371/journal.pcbi.1001028)
Supplement: Table S4 — Summary of Homo sapiens microarray data. (0.27 MB PDF) [file pcbi.1001028.s012.pdf]

Supplementary Table S4. Summary of *Homo sapiens* microarray data

Set	Accession Number	Description	Number of Samples	Reference
1	GSE8884	hESC differentiation into hemangioblasts	6	[1]
2	GSE9865	Reprograming of dermal fibroblasts	13	[2]
3	GSE7234	Expression profile of karyotypically abnormal hESC lines	4	-
4	GSE9086	hESC differentiation into hemangioblasts	13	[1]
5	GSE6561	Culture adaptation of hESCs	4	[3]
6	GSE9832	Reprograming of human somatic cells	16	[4]
7	GSE9709	hIPSCs from neonatal cells	13	[5]
8	GSE9940	Neural differentiation of hESCs	18	-
9	GSE7896	S1P mediated transcriptional regulation in hESCs	8	[6]
10	GSE12390	High-efficiency system for hIPSC generation	21	[7]
11	GSE12034	Transcriptome of human oocytes	6	[8]
12	GSE10809	SOX7 and SOX17 over-expressing hESC lines	8	[9]
13	GSE11350	hIPSCs from adult human testes	12	-

1. Lu SJ, Hipp JA, Feng Q, Hipp JD, Lanza R, et al. (2007) GeneChip analysis of human embryonic stem cell differentiation into hemangioblasts: an in silico dissection of mixed phenotypes. Genome Biol 8: R240.
2. Lowry WE, Richter L, Yachechko R, Pyle AD, Tchieu J, et al. (2008) Generation of human induced pluripotent stem cells from dermal fibroblasts. Proc Natl Acad Sci U S A 105: 2883-2888.
3. Baker DE, Harrison NJ, Maltby E, Smith K, Moore HD, et al. (2007) Adaptation to culture of human embryonic stem cells and oncogenesis in vivo. Nat Biotechnol 25: 207-215.
4. Park IH, Zhao R, West JA, Yabuuchi A, Huo H, et al. (2008) Reprogramming of human somatic cells to pluripotency with defined factors. Nature 451: 141-146.

5. Masaki H, Ishikawa T, Takahashi S, Okumura M, Sakai N, et al. (2007) Heterogeneity of pluripotent marker gene expression in colonies generated in human iPS cell induction culture. Stem Cell Res 1: 105-115.
6. Avery K, Avery S, Shepherd J, Heath PR, Moore H (2008) Sphingosine-1-phosphate mediates transcriptional regulation of key targets associated with survival, proliferation, and pluripotency in human embryonic stem cells. Stem Cells Dev 17: 1195-1205.
7. Maherali N, Ahfeldt T, Rigamonti A, Utikal J, Cowan C, et al. (2008) A high-efficiency system for the generation and study of human induced pluripotent stem cells. Cell Stem Cell 3: 340-345.
8. Kocabas AM, Crosby J, Ross PJ, Otu HH, Beyhan Z, et al. (2006) The transcriptome of human oocytes. Proc Natl Acad Sci U S A 103: 14027-14032.
9. Seguin CA, Draper JS, Nagy A, Rossant J (2008) Establishment of endoderm progenitors by SOX transcription factor expression in human embryonic stem cells. Cell Stem Cell 3: 182-195.
