# Supplementary material for: A Scalable Approach for Discovering Conserved Active Subnetworks across Species
Source: PLoS Comput Biol. 2010 Dec 9;6(12):e1001028. doi: 10.1371/journal.pcbi.1001028 (PMC3000367; doi:10.1371/journal.pcbi.1001028)
Supplement: Text S1 — This document contains the following supplementary notes: Note 1: Implications of using functional linkage vs. physical interaction networks for active subnetwork discovery; Note 2: neXus applied to single dataset differential expression study; Note 3: Independence of the datasets; Note 4: Comparison of the overlap of mouse and human subnetworks discovered through MATISSE and neXus; Note 5: Other randomizations. (0.42 MB PDF) [file pcbi.1001028.s014.pdf]

Supplementary Materials for

A Scalable Approach for Discovering Conserved Active Subnetworks across Species

Raamesh Deshpande et al.

Note 1: Implications of using functional linkage vs. physical interaction networks for active subnetwork discovery

While previous work has focused on subnetwork discovery in the context of physical (protein-protein) interaction networks, we propose here to use functional linkage networks, which are now available in a range of organisms from yeast to human. There are a number of implications of this decision, which we briefly discuss here. In general, protein-protein interactions reflect direct physical interactions between proteins, which means that they have the ability to capture stably bound protein complexes or potentially interactions that mediate signaling events. On the other hand, functional linkage networks are constructed by incorporating a much broader range of relationships: for example, physical interactions, co-expression, shared regulatory motifs, common protein localization patterns, or shared phylogenetic relationships. All of these are indicative of a function in a common biological process, and integration methods weight the input datasets according to their ability to predict known relationships, but the resulting linkages are less direct, and often less functionally specific. The broader relationships captured by functional linkage networks provide both unique advantages as well as additional challenges in the context of active subnetwork discovery.

In terms of advantages, they can potentially reveal broader functional modules or pathways which are not directly connected by physical protein-protein interactions, but are nonetheless functionally coherent and show similar patterns of differential expression. Protein-protein interaction networks will miss a large number of relationships captured by a functional linkage network, and thus, may not allow detection of the same coherently expressed modules. For example, it is not likely that transcription factors will show up in the same module as downstream targets in networks that truly reflect physical interactions between proteins, while this is common in subnetworks identified using functional linkage networks.

Functional linkage networks also introduce new challenges to the subnetwork discovery problem. Due to the generality of relationships captured by these networks, interpretation of the resulting active modules is often more difficult. They can include relationships based on a variety of underlying physical or genetic evidence, so even when coherent functions are represented among a set of genes, the underlying mechanisms supporting the putative relationships are not always clear. Each module must be investigated individually to assess the underlying evidence for the relationships before clear hypotheses can be formed. The higher density of functional linkage networks, which can be orders of magnitude more dense, may also present more technical problems in the discovery of active subnetworks. For example, the probability of finding dense subnetworks among even randomly chosen genes increases with the increased density of linkages. The higher density also adds complexity to the subnetwork discovery problem, which may require new algorithms as we discuss here.

In this work, we have chosen to use functional linkage networks as the basis of our approach because we feel that the added sensitivity to a broader range of functional relationships and availability of comprehensive functional linkage networks for several organisms, including human and mouse, are major advantages. Particularly when relatively independent expression data and networks are available in related species, the more comprehensive coverage offered by functional linkage networks can be a major advantage. Furthermore, given that our current understanding of physical protein-protein interactions is still limited in higher eukaryotes, functional relationships inferred from patterns in genomic data can offer a powerful approach to discovering new biology.

Note 2: neXus applied to single dataset differential expression study

The main contribution of this work is the cross-species subnetwork search algorithm, which is completely independent of the method for generating gene lists and activity scores. To illustrate the application of our algorithm, we compiled a large compendium of stem cell expression data for both mouse and human and derived a set of differentially expressed genes as described in Materials and Methods. However, to demonstrate that the search algorithm is independent of the differential expression analysis method, we also ran our cross-species search algorithm on gene lists derived from a simple application of SAM (Significance Array of Microarrays [1]) to a single mouse expression dataset (GSE 3653) [2] and a single human expression dataset (GSE 9940) [3]. We also randomized the resulting expression values and searched for subnetworks on the randomized data for comparison (Figure S1). The conclusion from this analysis is similar to that from the analysis of our original differential expression list: our approach is able to find many significant subnetworks from the real differential expression list but very few based on the randomized differential expression data. For example, at an activity score cutoff of 0.2, our approach discovers 48 subnetworks on the real differential expression values but an average of 2 on the randomized data (Figure S1).

Note 3: Independence of the datasets

An important issue that affects the significance of active subnetworks discovered is the independence of the various input datasets, including the relationship between the differential expression data and the functional networks within each species as well as the relationship between the functional networks across species. Because expression data is one of the major sources of input data for constructing both the mouse and human functional linkage networks, we checked whether the datasets we compiled for our stem cell differential expression analysis overlapped with those used in constructing the mouse and human functional linkage networks. None of the 20 mouse datasets (Supplemental Table S3) or 13 human datasets (Supplemental Table S4) used for our differential expression analysis were used to construct the mouse or human functional linkage networks. Thus, these data are independent. With regard to the independence of the mouse and human functional linkage network, the mouse network was constructed first (2008), and was not used as input in constructing the human functional linkage network. The human network incorporates physical and genetic interactions, sequence information (shared protein domains, transcription factor binding sites), and gene expression profiles [4]. The mouse network incorporates physical interaction data, shared phenotype data, phylogenetic profile information, the yeast functional network where orthologs exist, and gene expression information [5]. Both resulted from naïve Bayes classifiers that were trained using Gene Ontology annotations, which are

of course not independent between mouse and human, but we would argue that this is also true of any interaction network available for mouse or human, so it is not an easy issue to avoid. Another source of dependence between the mouse and human network is that the mouse functional network incorporates putative protein interactions from the Online Predicted Human Interaction Database (OPHID), which were mapped from human orthologs (commonly referred to as interologs). OPHID itself was not directly used in constructing the human functional linkage network, but is based on several of the protein-protein interactions that were. Thus, the physical interaction data incorporated in the mouse network are not independent of the physical interactions incorporated in the human network. For our analysis in this paper, we have chosen to keep the mouse network as originally constructed because of the limited availability of mouse-specific interactions. We should note this dependence between the two functional networks is accounted for in the randomization procedure, which we used to statistically validate our results (see Figure 2). For all randomization experiments, the functional linkage networks were held fixed in both species (only the differential expression values are randomized), so whatever dependence exists should also help increase the number of networks discovered during random instances. Our approach recovers ~20-fold more subnetworks than the average random run (see Figure 2), suggesting that the algorithm is accomplishing something useful even if the human and mouse functional networks are not completely independent.

Note 4: Comparison of the overlap of mouse and human subnetworks discovered through MATISSE and neXus

To check whether single-species approaches could be used to discover conserved active subnetworks, we applied MATISSE [6], the existing method for single-species network discovery with the best performance (see Results, Figure 4). Both approaches were applied independently to the mouse and human differential expression data and functional linkage networks. As discussed in the Results, we were not able to apply MATISSE to the complete mouse or human functional linkage network, so we reduced the size of the networks to the size where we could load and run the algorithm on the networks, which was to 50,000 edges (7909 genes) and 25,000 edges (9281 genes) for mouse and human, respectively. For both, the edges were restricted to the highest weight edges. We then ran MATISSE independently on these reduced networks and the corresponding expression profiles. Mouse subnetworks and human subnetworks were then compared for overlap to assess how it compared to our cross-species algorithm. In terms of the number of genes, the mouse and human subnetworks covered around three thousand genes (Table S1), roughly half of which were orthologs (Table S1). However, the MATISSE subnetworks showed relatively low agreement in terms of the sets of genes present. Only a single pair was found to have a Jaccard index ($A \cap B / A \cup B$) greater than 0.2, suggesting that even where orthologs overlap, the sets of modules discovered in human and mouse are quite distinct (Table S2). In contrast, our algorithm is designed to find completely overlapping subnetworks. Thus, we find nearly 100% overlap in terms of the genes covered in our approach, and all subnetworks have a Jaccard index near 1 (Table S2). We should note that this is not surprising given the fact that our search algorithm identifies networks on orthologous genes in parallel, but this result does demonstrate the utility of this type of search in the sense that it enables a more direct comparison of the human and mouse expression patterns

Note 5: Other Randomizations

In addition to the randomization scheme described in the Results section, which involves shuffling the the differential expression values in both species, we evaluated three other schemes as well: randomizing differential expression values in only mouse, randomizing differential expression values in only human, and randomizing the orthology links between mouse and human. At the same parameters at which we discover 255 real subnetworks (mouse and human clustering coefficient = 0.1 and 0.2 respectively and network score cutoff = 0.15), we found an average of ~11 with our original randomization approach, an average of ~30 with the mouse-only randomization, an average of ~24 with the human-only randomization, and an average of ~3 with the orthology randomization (Figure S8). Even by the most conservative randomization scheme, our approach finds ~10-fold more real networks than random

References

1. Tusher VG, Tibshirani R, Chu G (2001) Significance analysis of microarrays applied to the ionizing radiation response. Proc Natl Acad Sci U S A 98: 5116-5121.
2. Treff NR, Vincent RK, Budde ML, Browning VL, Magliocca JF, et al. (2006) Differentiation of embryonic stem cells conditionally expressing neurogenin 3. Stem Cells 24: 2529-2537.
3. Zhang S (2008) ES cells, EBs grown in suspension (d6), PEL (d10) stage and neural rosettes (d17) (zhang-affy-human-346640). Apr 19, 2008 ed: NCBI Gene Expression Omnibus.
4. Huttenhower C, Haley EM, Hibbs MA, Dumeaux V, Barrett DR, et al. (2009) Exploring the human genome with functional maps. Genome Res 19: 1093-1106.
5. Guan Y, Myers CL, Lu R, Lemischka IR, Bult CJ, et al. (2008) A genomewide functional network for the laboratory mouse. PLoS Comput Biol 4: e1000165.
6. Ulitsky I, Shamir R (2007) Identification of functional modules using network topology and high-throughput data. BMC Syst Biol 1: 8.
